# Genome-wide association study identifies novel genes for plant architecture and yield traits in cassava (*Manihot esculenta* Crantz)

**DOI:** 10.3389/fpls.2025.1660789

**Published:** 2025-09-10

**Authors:** Abiodun Fatai Olayinka, Daniel Kwadjo Dzidzienyo, Edwige Gaby Nkouaya Mbanjo, Samuel Kwame Offei, Pangirayi Bernard Tongoona, Eric Yirenkyi Danquah, Chiedozie Egesi, Ismail Yusuf Rabbi

**Affiliations:** ^1^ West Africa Centre for Crop Improvement (WACCI), College of Basic and Applied Sciences, University of Ghana, Accra, Ghana; ^2^ International Institute of Tropical Agriculture (IITA), Ibadan, Nigeria; ^3^ Biotechnology Centre, College of Basic and Applied Sciences, University of Ghana, Accra, Ghana; ^4^ Cassava Research Program, National Root Crops Research Institute (NRCRI), Umudike, Nigeria; ^5^ Department of Plant Breeding and Genetics, Cornell University, Ithaca, NY, United States

**Keywords:** SNP markers, *Manihot esculenta* Crantz, mechanical farming, DArTseq, DArTag, novel genes

## Abstract

Cassava (*Manihot esculenta* Crantz) cultivars with compact plant types and moderate plant heights are required for mechanical farming to boost productivity. Plant architecture is a complex trait controlled by environmental and genetics factors. However, little is known about the genetic basis of cassava plant architecture. This research sought to bridge the knowledge gap by elucidating the genetic basis of traits related to plant architecture, yield, and productivity in cassava. A panel of 453 cassava clones developed at the International Institute of Tropical Agriculture was genotyped using two distinct genotyping platforms: low-density DArTseq and DArTag. Plant architecture, yield, and productivity-related traits were evaluated at three locations across two growing seasons in Nigeria. Following data filtering, 420 clones, 54,574 DArTSeq, and 2,527 DArTag single-nucleotide polymorphism (SNP) markers were used for genome-wide association studies (GWAS). Of the 16 SNPs identified by GWAS using DArTSeq markers, only one was detected during validation, and the remaining SNPs may be false positives. Sixteen SNPs were found to be significant using DArTag markers. Fifteen of these were associated with 21 putative candidate genes for five plant architecture traits (17 genes) and three yield traits (four genes). Six of the identified candidate genes were novel. The identified candidate genes were associated with various metabolic processes, including plant architecture, adaptation, root development, plant growth, and stress response. The limited number of significant markers identified using DArTSeq markers could be explained by the large gaps and uneven marker distribution observed across the genome with the DArTseq platform compared to DArTag. The findings of this study provide new insights into the genetic basis of plant architecture and yield in cassava. Cassava breeders could leverage this knowledge to optimize plant architecture and yield in cassava through marker-assisted selection and targeted manipulation of candidate genes.

## Introduction

Cassava is Africa’s second most important food crop. Over 40% of the continent’s population relies on it to fulfil their daily caloric and nutritional needs (Alves, 2002; Nweke, 2004; FAO, 2020). Cassava starch is a versatile raw material for food, feed, and industrial applications (Li et al., 2017). Despite the huge prospects of its value chain, cassava production in Nigeria remains low at approximately 10 tons per hectare, while demand is increasing (FAO, 2021). The increasing demand for cassava-based products necessitates the drive to increase crop performance to boost productivity.

Plant architecture plays a crucial role in agriculture. It affects crop growth, yield, and stress resistance (Lauri and Lespinasse, 2001; Rymaszewski et al., 2017; Mansaray et al., 2020). Cassava cultivars with appropriate plant architecture are needed to maximize cassava yield potential, mechanize cassava farming, and boost productivity per unit area (Patiño et al., 2002; FAO, 2013). Plant architecture and yield are complex traits known to be controlled by both environmental and genetic factors (Zhang et al., 2017; Yu et al., 2020). Several studies have attempted to dissect the genetic basis of cassava plant architecture-related traits and their effects on cassava yield using biparental populations, with limited breakthroughs (OkogBenin and Fregene, 2003; Mora Moreno et al., 2016; Srisawad et al., 2023).

Genome-wide association studies (GWAS), which use a diversity panel, are gaining popularity for marker-trait associations (Dash et al., 2021; Uffelmann et al., 2021). GWAS has helped unravel the genetic architecture of important crop plant traits, including yield and quality traits, early bulking and storage root formation, and nitrogen use efficiency in cassava (Abah et al., 2024; Aghogho et al., 2024; Mbe et al., 2024), plant architecture in maize (Lu et al., 2024), and drought tolerance in wheat (Yang et al., 2024). Single-nucleotide polymorphisms (SNPs) have been the marker of choice for GWAS analyses owing to their stable inheritance patterns, low mutation rates, and compatibility with high-throughput genotyping technologies (Morgil et al., 2020; Panahi et al., 2024).

The reliability and effectiveness of GWAS could be influenced by the density and distribution of SNP markers throughout the genome (Spindel et al., 2015; Uffelmann et al., 2021; Aalborg et al., 2024). Information on the impact of marker density and distribution on GWAS output is limited. Two genotyping platforms, DArTseq and DArTag, were used to conduct GWAS analyses. This study aimed to investigate the genetics of plant architecture and yield-related traits in cassava, as well as how marker density and distribution affect GWAS effectiveness and reliability.

## Materials and methods

The cassava genotypes used in this study included 453 genotypes from a preliminary yield trial (PYT) developed at the IITA and five commercial varieties used as checks. The details of the accessions, crosses, and parents are reported in **Supplementary Table S1**. Descriptions of the commercial check varieties are provided in **Supplementary Table S2**. The trial was conducted in three locations in Nigeria: Ikenne (Lat 6.8718° N, Long 3.7106° E, humid forest zone), Onne (Lat 4.7363° N, Long 7.1545° E, humid forest zone), and Mokwa (Lat 9.2934° N, Long 5.0493° E, Southern Guinea Savannah zone) across two planting seasons (2020/2021, and 2021/2022). The trial was laid out in an augmented block design with two replications per location for each season. Each plot comprised three rows with three plants planted on ridges per row. A spacing of 1m was maintained between ridges, while a spacing of 0.8m was observed within plants in a row.

### Phenotypic data collection

Ten above-ground plant traits: stem diameter (STMDI9), shoot weight (SHTWT), plant height at 9 months after planting (PLTHT9), number of lodged plants per plot (LODG), branching habit at 9 months after planting (BRNHB9), angle of branching (ANGBR9), number of plants per stand (PPSTD9), height at first branch at 9 months after planting (BRNHT9), plant height at 6 months after planting (PLTHT6), top yield (TYLD), height at first branch at 6 months after planting (BRNHT6), and eight yield-related traits: fresh root yield (FYLD), dry yield (DYLD), starch content (SC), dry matter content (DM), number of harvested roots (RTNO), fresh root weight (RTWT), and harvest index (HI), were measured at specific phenological stages of the crop (3, 6, 9, and 12 months after planting). The phenological stage and method used to assess the traits were based on the parameters described by (Fukuda et al., 2010). The details of the traits evaluated and the evaluation procedures are presented in **Supplementary Table S3**.

### SNP genotyping and marker filtering

Leaf samples were collected from a single plant per cassava clone, 4–6 leaf discs of 6mm in diameter were collected from healthy young leaves. The leaf discs were placed in the corresponding wells of the DNA plate on ice in a sampling bag. The plate was immediately transferred to the laboratory, where it was covered with Parafilm and kept at -80°C in a freezer prior to freeze-drying. The samples were freeze-dried at the IITA Bioscience Centre using a lyophilizer (LABCONCO FreeZone 18 Liter -50°C freeze-dryer) operated at -51°C and 5.0 pa for a minimum of 72 hrs. The freeze-dried leaf samples were shipped to Diversity Arrays Technology in Canberra, Australia, where genotyping was performed using low-density DArTseq and DArTag technology. Upon receipt, the raw SNP data were filtered using PLINK 1.9 software (Purcell et al., 2007; Chang et al., 2015). SNP markers with less than 5% minor allele frequency (MAF) of more than 10% missing call rate were pruned.

### Statistical analyses of phenotypic data

Plant architecture and yield-related traits were analyzed using the spatial single-trial model fitted using the R package `SpATS` (Rodriguez-Alvarez et al., 2018). The mathematical formula for the adopted model is as follows:


$$y_{ijk}=\mu +g_{i}+r_{j}+b_{k}+s(x_{ijk},\;y_{ijk})+e_{ijk}$$

where y_ijk_ is the phenotypic value of the i^th^ genotype in the j^th^ block and k^th^ incomplete block, μ is the overall mean, g_i_ is the random effect of the i^th^ genotype, r_j_ is the random effect of the j^th^ block, b_k_ is the random effect of the k^th^ incomplete block, s(x_ijk_, y_ijk_) is the smooth bivariate function of the row and column coordinates of the plot, and e_ijk_ is the residual error. The best linear unbiased prediction (BLUP) values derived from this model were used as phenotypic values in the marker-trait association analysis.

The linear mixed model for the analysis of the pooled data (obtained from the three test environments) was fitted as follows using the lmer function from the lme4 package (Bates et al., 2015) of the R software (R Core Team, 2020).


$$\eta_{ijklm}=\mu +\alpha_{i}+\beta_{ij}+\gamma_{k}+\delta_{ik}+\epsilon_{ijl}+e_{ijkl}$$

where η_ijklm_ is the yield value for the m^th^ plot in the l^th^ block of the j^th^ replicate of the i^th^ environment for the k^th^ accession; μ is the overall mean; α_i_ is the fixed effect of the i^th^ environment; β_ij_ is the fixed effect of the interaction between the ith environment and the j^th^ replicate; γ_k_ is the random effect of the k^th^ accession; δ_ik_ is the random effect of the interaction between the i^th^ environment and the k^th^ accession; ϵ_ijl_ is the random effect of the interaction between the i^th^ environment, the j^th^ replicate, and the l^th^ block; and e_ijkl_ is the random residual error.

The random effects γ_k_, δ_ik_, and ϵ_ijl_ are assumed to be independent and normally distributed with a mean of 0 and variance components σ^2^a, σ^2^ea, and σ^2^b, respectively. The residual error eijkl is also assumed to be independent and normally distributed with a mean of 0 and variance component σ^2^e.

The best linear unbiased prediction (BLUP) values derived from this model were used as the pooled phenotypic values across environments. The broad-sense heritability (H^2^) values were calculated as follows:


$$H^{2}=\frac{\sigma_{g}^{2}}{\sigma_{g}^{2}+\frac{\sigma_{ge}^{2}}{e}+\frac{\sigma_{e}^{2}}{er}}$$

Where H^2^ is the broad-sense heritability estimate, σ^2^g is the genetic variance component of the accession effect, σ^2^e​ is the variance component of the residual error, and r is the number of replicates.

Genotypic correlation analysis for the plant architecture and yield-related traits was performed using the statistical software Meta-R Version 6.0 (Alvarado et al., 2020). Variance components were estimated through a mixed linear model fitted using Restricted Maximum Likelihood (REML) to obtain estimates of genotypic variance, variance due to genotype-by-environment interaction, and residual variance prior to the computation of the genotypic correlations among traits.

The formula for the mixed linear model is as follows:


$${\text{y}}_{\text{ijk}}=\text{μ}+{\text{α}}_{\text{i}}+{\text{β}}_{\text{j}}+{\left(\text{αβ}\right)}_{\text{ij}}+{\text{γ}}_{\text{k}}+{\text{e}}_{\text{ijk}}$$

y_ijk_ is the observation for the i-^th^ genotype in the j-^th^ block and the k-^th^ replication

μ is the overall mean

α_i_ is the fixed effect of the i-^th^ genotype

β_j_ is the random effect of the j-^th^ block

(αβ)_ij_ is the random interaction effect of the i-^th^ genotype and the j-^th^ block

γ_k_ is the random effect of the k-^th^ replication within the block

e_ijk_ is the residual error.

The estimates of the genetic correlation coefficient values were obtained using the variance components from the mixed linear model:


y=Xβ+Zu+e

y is the vector of observations

β is the vector of fixed effects

u is the vector of random effects

e is the vector of residuals

X and Z are design matrices.

The genetic correlation between the two traits was calculated as follows:


$$rg=\frac{\sigma_{g1g2}}{\sqrt{\sigma_{g1}^{2}\sigma_{g2}^{2}}}$$

σg_1_g_2_ is the covariance between the random effects of the two traits. σ^2^g_1_ and σ^2^g_2_ are the variances of the random effects of the two traits, respectively. Data visualization was performed with the R package ‘corrplot’ version 0.92 (Wei and Simko, 2021). Path Analysis was performed using the R package ‘lavaan’ version 0.6-17 (Rosseel, 2012) and visualized using the R package ‘semPlot’ (Epskamp et al., 2019).

### Marker coverage, SNP density, and linkage disequilibrium

Marker coverage and SNP density were visualized using the SRplot platform (Tang et al., 2023). Linkage Disequilibrium (LD) analysis was conducted using PLINK v1.9 (Purcell et al., 2007). Marker pairs exhibiting perfect LD scores (r^2^=1) were excluded before further analysis. An LD decay plot was created utilizing R software (R Core Team, 2020).

### Population structure analysis

Population structure analysis was performed using ADMIXTURE (Alexander et al., 2009), which uses the maximum likelihood estimate to assign genotypes to putative populations (K). Cross-validation was performed to determine the optimal number of clusters. The results of the population structure analysis were visualized using the R package “ggplot2” (Wickham, 2016). Ward’s minimum variance method (ward.D2) was utilized for hierarchical clustering on the Q-matrix produced by ADMIXTURE. The R package “dendextend” (Galili, 2015) was used to plot the dendrogram.

### Genetic association analysis

GWAS analysis was performed with the BLUP values obtained from the trials conducted in each of the three test environments as well as the pooled data across all the environments through the Mixed Linear Model (MLM) described in (Zhang et al., 2010), using the R package Genome Association and Prediction Integrated Tools (GAPIT) (Lipka et al., 2012). This model decomposes the observed phenotype (y) into fixed effects (Xβ), random genetic effects (Zu), and residual effects (e). The formula is:


y=Xβ+Zu+e

where X is the design matrix for fixed effects, Z is the design matrix for random effects, β is the vector of fixed effects, u is the vector of random effects, and e is the vector of residuals.

In addition to the MLM model, the Fixed and random model Circulating Probability Unification (FarmCPU) model outlined in (Liu et al., 2016) was used for GWAS carried out using DArTseq markers. FarmCPU uses a modified MLM method, Multiple Loci Mixed Model (MLMM), and incorporates multiple markers simultaneously as covariates in a stepwise MLM to partially remove the confounding effects between testing markers and kinship. The formula is:


$$\text{y}=\text{Xβ}+{\text{Z}}_{1}{\text{u}}_{1}+{\text{Z}}_{2}{\text{u}}_{2}+\text{e}$$

where X is the design matrix for fixed effects, Z_1_ is the design matrix for random effects from kinship, Z_2_ is the design matrix for random effects from multiple markers, β is the vector of fixed effects, u_1_ is the vector of random effects from kinship, u_2_ is the vector of random effects from multiple markers, and e is the vector of residuals.

These models deployed different approaches in accounting for the effects of population structure and environmental variation thereby precluding any possibility of false discovery. However, the following criteria were used in calling SNPs that share a significant correlation with the plant architecture and yield traits: Bonferroni correction, the threshold of the P-value, Manhattan plot and the QQ plot.

The Bonferroni-corrected p-value [log10(0.05/number of SNPs)] was used as a cutoff value for identifying significant SNPs. Manhattan plots were generated to visualize the results, while quantile-quantile (QQ) plots showed the distribution of observed p-values against those predicted under the null hypothesis and captured potential inflation or deflation of the test statistic. Additionally, we used the mixed linear model, which excludes (MLMe) and includes (MLMi) candidate markers in the genetic relationship matrix (GRM) via a leave-one chromosome-out analysis implemented in GCTA (Yang et al., 2011; Yang et al., 2014). The exclusion of the tested SNP from the relationship matrix (GRM) used in the random effect reduces proximal contamination or overcorrection, thereby preventing deflating associations on the same chromosome as the tested SNP. This helped verify the authenticity of the significant SNPs identified using the other two models.

### Candidate gene analysis

The significant SNPs were mapped onto genes within the 5,000 bp windows using *Manihot esculenta* v7.1 of the Phytozome genome browser (Goodstein et al., 2014). The UniProt Consortium database (Aleksander et al., 2023) was used for gene ontology annotation.

## Results

### Marker coverage and SNP density

**Figure 1:**
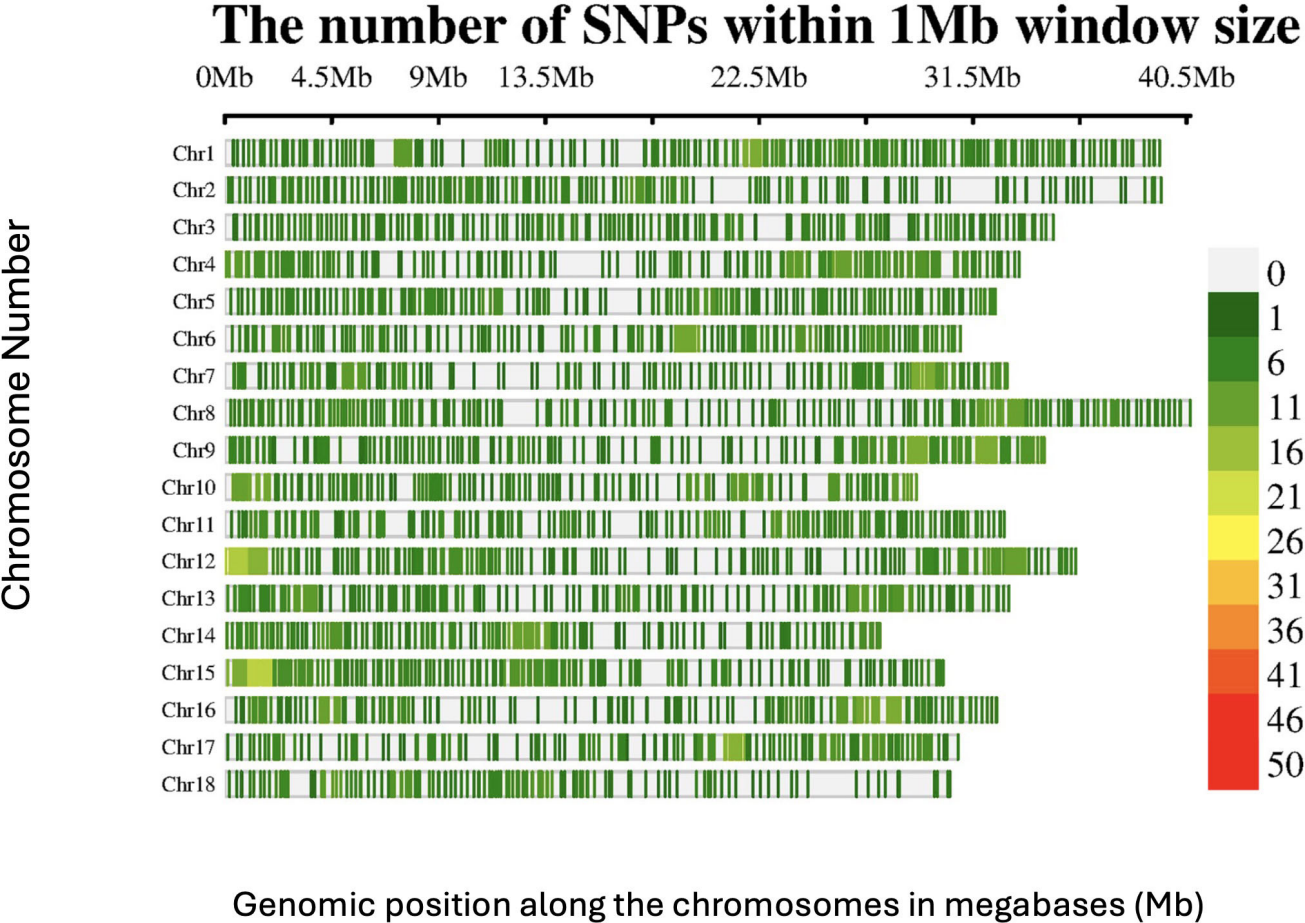
SNP distribution of the DArTAG markers across the 18 chromosomes of the *Manihot esculenta* genome.

A total of 2,527 DArTag SNP markers, uniformly distributed over the 18 chromosomes, were retained after marker filtering (**Figure 1**). The average number of SNPs per 1MB window across the genome ranged from 1 to 26 SNPs for most of the genomic regions. Chromosome 8 had the highest number of SNPs (164), whereas chromosome 18 had the lowest (106).

**Figure 2:**
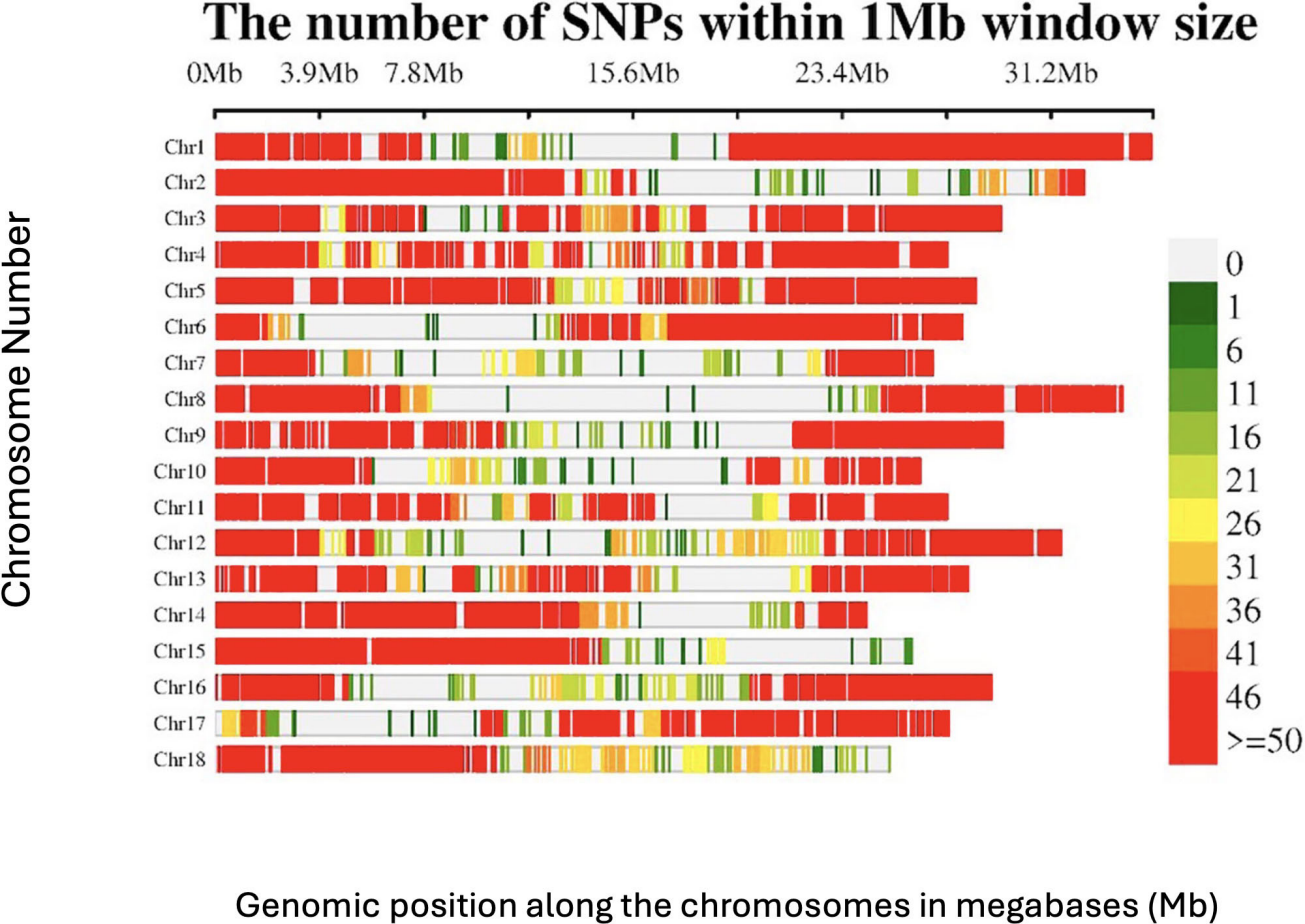
SNP distribution of the DArTSeq markers across the 18 chromosomes of the *Manihot esculenta* genome.

The 54,574 filtered DArTSeq SNP markers were not evenly distributed across the 18 chromosomes (**Figure 2**). Some genomic regions, especially those close to the centromeric and telomeric regions, showed very low SNP densities, and in some cases, no markers, whereas other regions displayed a dense clustering of SNPs (>50 SNPs per 1 Mb window). Chromosome 1 had the highest number of SNPs (7235), with an average of 207 SNPs/Mb, whereas chromosome 7 had the fewest (1588), with approximately 59 SNPs/Mb.

### Linkage disequilibrium

**Figure 3:**
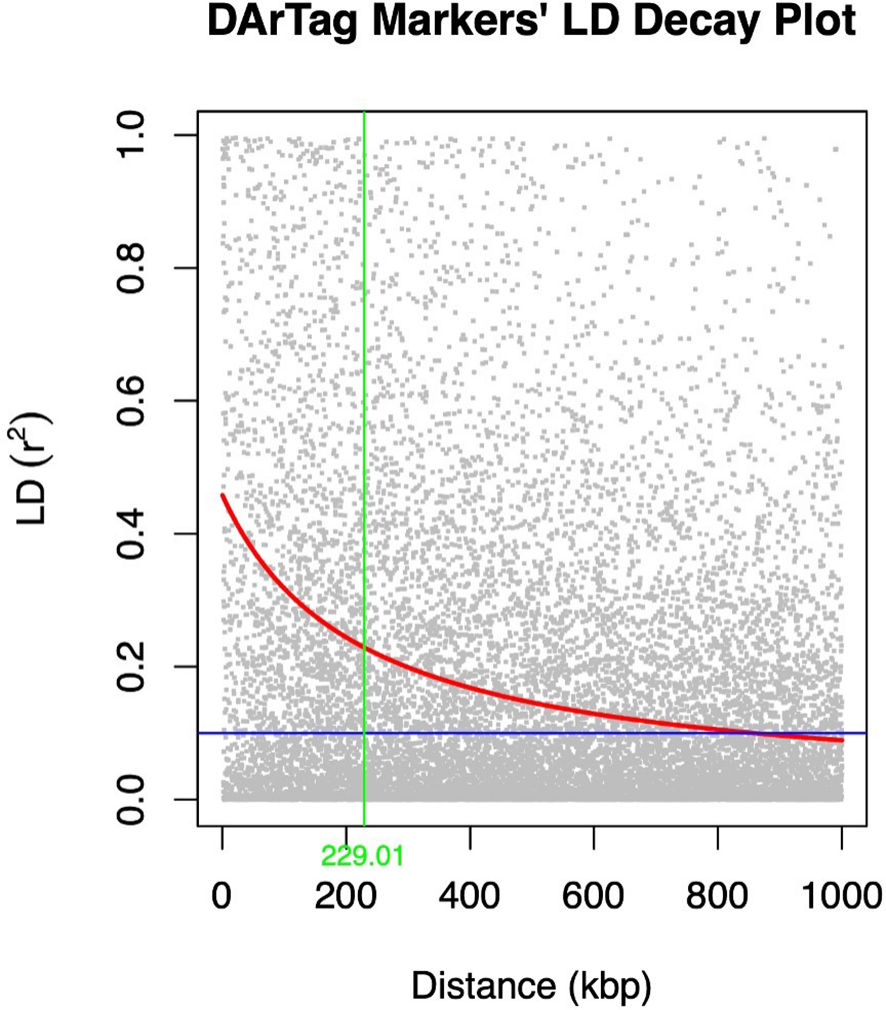
LD decay plot of the DArTag markers generated using PLINK and visualized in R.

**Figure 4:**
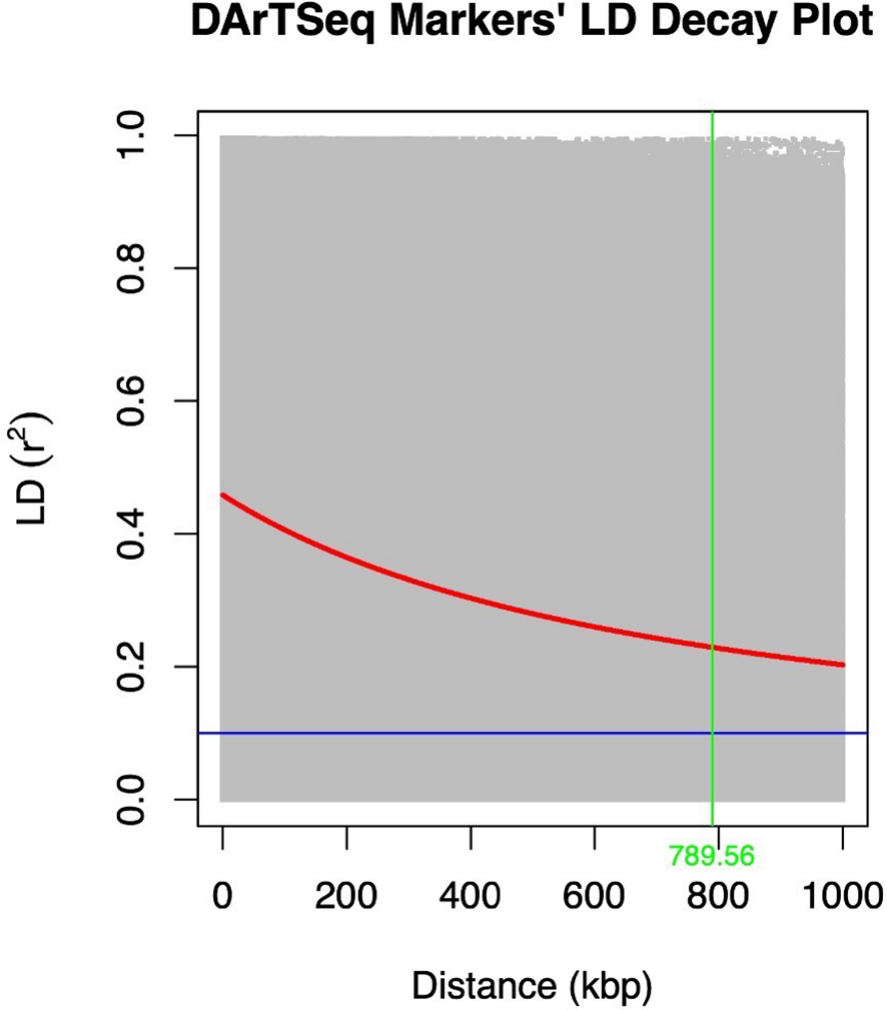
LD decay plot of the DArTSeq markers performed with PLINK and visualized in R.

The extent of LD decay was compared between the two marker types used (**Figures 3**, **4**). A rapid LD decay was observed using DArTag markers. The LD between two SNPs fell to 0.2 (the standard threshold that indicates the end of LD) at a relatively more rapid rate, approximately 229 kbp (**Figure 3**), contrary to the output obtained with the DArTSeq markers (**Figure 4**), where the LD between two SNPs fell to 0.2 at a longer distance of approximately 790 kbp.

### Population structure analysis

**Figure 5:**
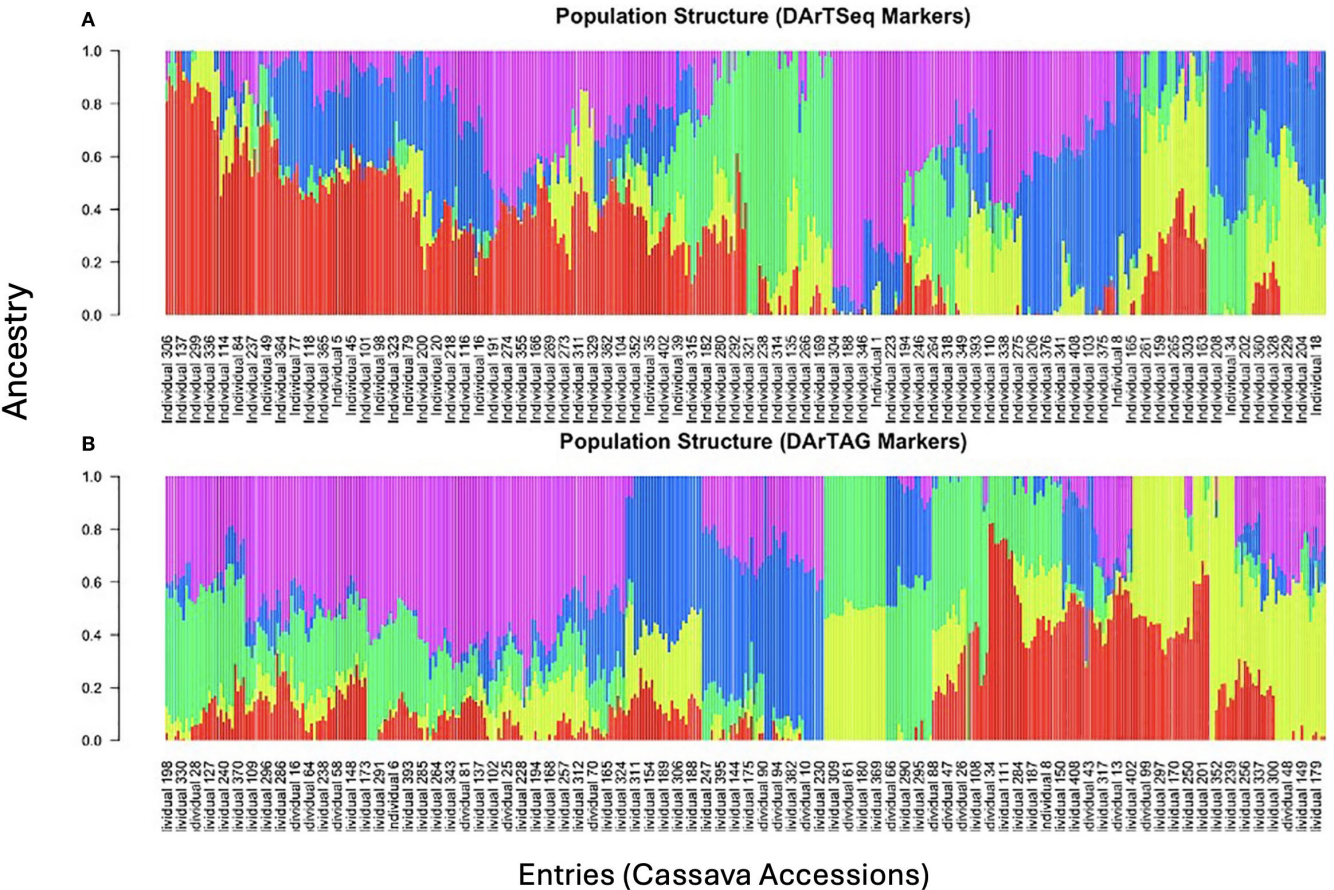
Population structure within the cassava accessions for DArTSeq markers and DArTag markers.

Population structure analyses revealed five subgroups (**Figure 5**). The cassava accessions were developed from crosses made using parents with diverse genetic backgrounds (**Supplementary Table S1**). The plot also shows that there is a balance between the extent of admixture and homogeneity within the population. This shows that there exists enough genetic diversity within the population to capture the variation in the plant architecture and yield traits.

### Variance Components and broad-sense heritability of plant architecture and yield-related traits

All 18 traits exhibited greater genotypic variance than both the error variance and the variance due to the interaction between genotype and environment (**Table 1**). Both genetic and interaction factors had a strong influence on the performance of the accessions for all measured traits (p< 0.001). The broad-sense heritability varied from moderately high (0.62; SHTWT) to high (0.88; BRNHT9). The broad-sense heritability for key plant architecture traits, viz., ANGBR9 (0.78), STMDI9 (0.73), PLTHT9 (0.80), PLTHT6 (0.83), and BRNHT6 (0.86) were found to be high, while the following yield-related traits, including DM (0.68), SC (0.70), FYLD (0.67), and HI (0.86) had relatively high broad-sense heritability values (**Table 1**). The phenotypic and genotypic coefficients of variation ranged from 8.30% and 6.84% (DM), respectively, to 78.28% and 65.03% (LODG) (**Table 1**).

**Table 1:** Estimates of variance components and broad-sense heritability for plant architecture and yield-related traits in 438 cassava accessions evaluated across three locations in Nigeria.

Trait	Mean	σ^2^g	σ^2GxE^	σ^2^e	H^2^	PCV(%)	GCV(%)
STMDI9	24.00	5.56	0.81	1.28	0.73	11.52	9.82
FYLD	23.00	37.20	9.00	9.32	0.67	32.40	26.52
DYLD	7.80	3.70	0.97	1.08	0.64	30.74	24.66
SC	19.80	5.84	1.71	0.85	0.70	14.63	12.21
DM	32.60	4.97	1.53	0.80	0.68	8.29	6.84
RTWT	19.20	25.50	6.67	6.43	0.66	32.36	26.30
SHTWT	21.00	21.80	4.73	8.50	0.62	28.19	22.23
PLTHT9	176.90	507.80	40.83	84.67	0.80	14.23	12.74
HI	0.47	0.01	0.00	0.00	0.86	22.98	21.28
BRNHB9	2.60	0.17	0.04	0.03	0.71	18.78	15.86
ANGBR9	81.00	60.70	6.00	10.93	0.78	10.88	9.62
RTNO	44.60	131.40	29.87	41.58	0.65	31.93	25.70
PPSTD9	2.00	0.10	0.01	0.05	0.63	19.90	15.81
BRNHT9	83.80	580.00	33.63	48.00	0.88	30.69	28.74
PLTHT6	158.00	459.00	30.57	64.67	0.83	14.90	13.56
TYLD	25.90	31.50	6.40	13.17	0.62	27.59	21.67
BRNHT6	74.00	477.60	36.00	44.93	0.86	31.94	29.53
BRLEV9	2.30	0.34	0.06	0.03	0.78	28.62	25.35

**Figure 6:**
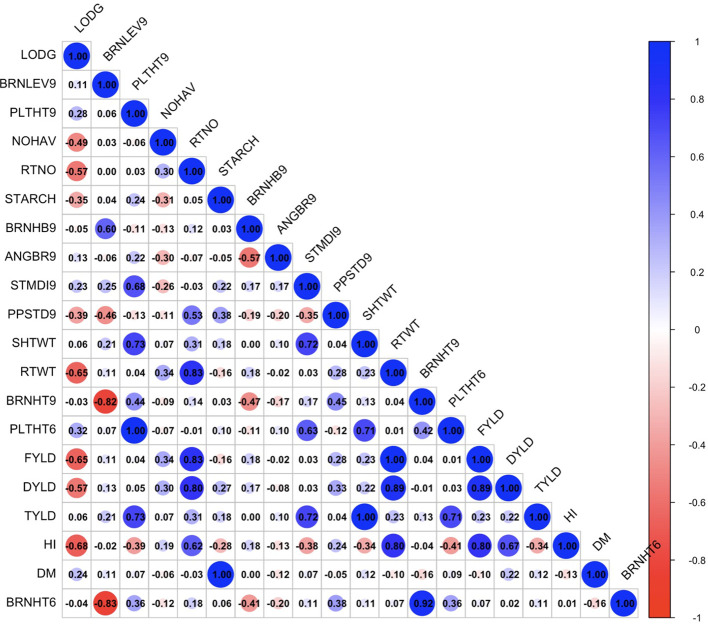
Genotypic correlation coefficient plot of plant architecture and yield traits in Ikenne trial. NOHAV, number of harvested plants per plot; RTNO, number of harvested roots per plot; SHTWT, shoot weight; SC, starch content; FYLD, fresh root yield; DYLD, dry yield; HI, harvest index; LODG, number of lodged plants per plot; PLTHT6, plant height at 6 months after planting; BRNHT6, height at first branch at 6 months after planting; PLTHT9, plant height at 9 months after planting; BRNHT9, height at first branch at 9 months after planting; BRNLEV9, level of branching at 9 months after planting; BRNHB9, branching habit at 9 months after planting; ANGBR9, angle of branching; PPSTD9, number of plants per stand, and STMDI9, stem diameter.

**Figure 7:**
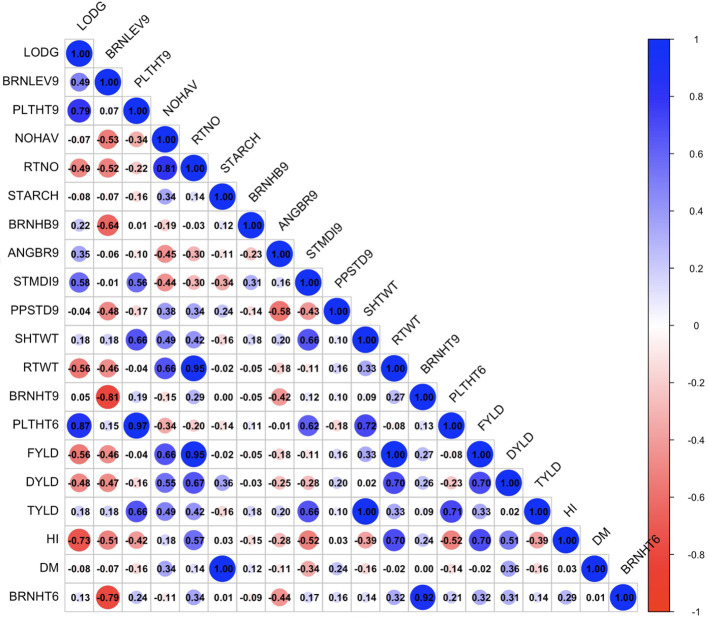
Genotypic correlation coefficient plot of plant architecture and yield traits in Mokwa trial. NOHAV, number of harvested plants per plot; RTNO, number of harvested roots per plot; SHTWT, shoot weight; SC, starch content; FYLD, fresh root yield; DYLD, dry yield; HI, harvest index; LODG, number of lodged plants per plot; PLTHT6, plant height at 6 months after planting; BRNHT6, height at first branch at 6 months after planting; PLTHT9, plant height at 9 months after planting; BRNHT9, height at first branch at 9 months after planting; BRNLEV9, level of branching at 9 months after planting; BRNHB9, branching habit at 9 months after planting; ANGBR9, angle of branching; PPSTD9, number of plants per stand; STMDI9, stem diameter.

**Figure 8:**
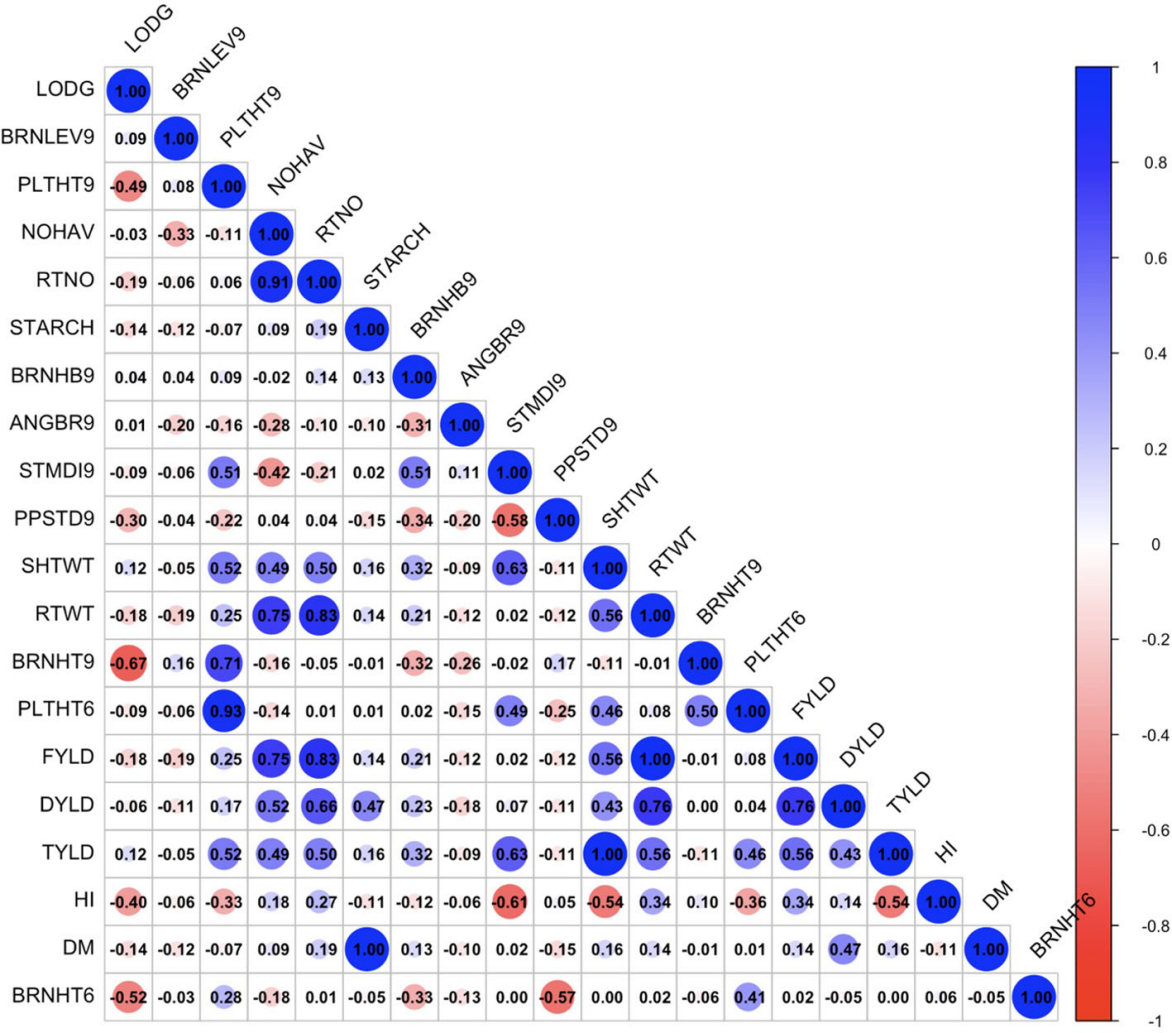
Genotypic correlation coefficient plot of plant architecture and yield traits in Onne trial. NOHAV, number of harvested plants per plot; RTNO, number of harvested roots per plot; SHTWT, shoot weight; SC, starch content; FYLD, fresh root yield; DYLD, dry yield; HI, harvest index; LODG, number of lodged plants per plot; PLTHT6, plant height at 6 months after planting; BRNHT6, height at first branch at 6 months after planting; PLTHT9, plant height at 9 months after planting; BRNHT9, height at first branch at 9 months after planting; BRNLEV9, level of branching at 9 months after planting; BRNHB9, branching habit at 9 months after planting; ANGBR9, angle of branching; PPSTD9, number of plants per stand; STMDI9, stem diameter.

The exploratory analysis revealed the interrelationships between plant architecture and yield-related traits. The genetic correlation coefficient matrix revealed positive and significant relationships between FYLD and DYLD (r = 0.70 – 0.89, p < 0.001), PLTHT6 and PLTHT9 (r = 0.93 – 1.00, p < 0.001), STDMI9 and PLTHT6 (r = 0.49 – 0.63, p < 0.001), and STDMI9 and PLTHT9 (r = 0.51 – 0.68, p < 0.001) (**Figures 6**–**8**). Negative and significant relationships were observed between BRNHT6 and BRNLEV9 (r = -0.03 – (-0.83), p < 0.001) and BRNHT9 and BRNLEV9 (r = -0.81 – (-0.82), p < 0.001) (**Figures 6**–**8**). In addition to these, significant negative relationships were observed between LOGD and FYLD (r = -0.18 - (-0.65), p < 0.001), RTNO (r = -0.19 – (-0.57), PPSTD9 (r = -0.04 - (-0.30), p < 0.001) and HI (r = -0.40 – (-0.73), p < 0.001) respectively. Similarly, there was a strong negative and significant correlation between HI and some crucial plant architecture traits, viz., STMDI9 (r = -0.38 – (-0.61), p < 0.001), PLTHT6 (r = -0.36 – (-0.52), p < 0.001), and PLTHT9 (r = -0.33 – (-0.42), p < 0.001) (**Figures 6**–**8**).

**Figure 9:**
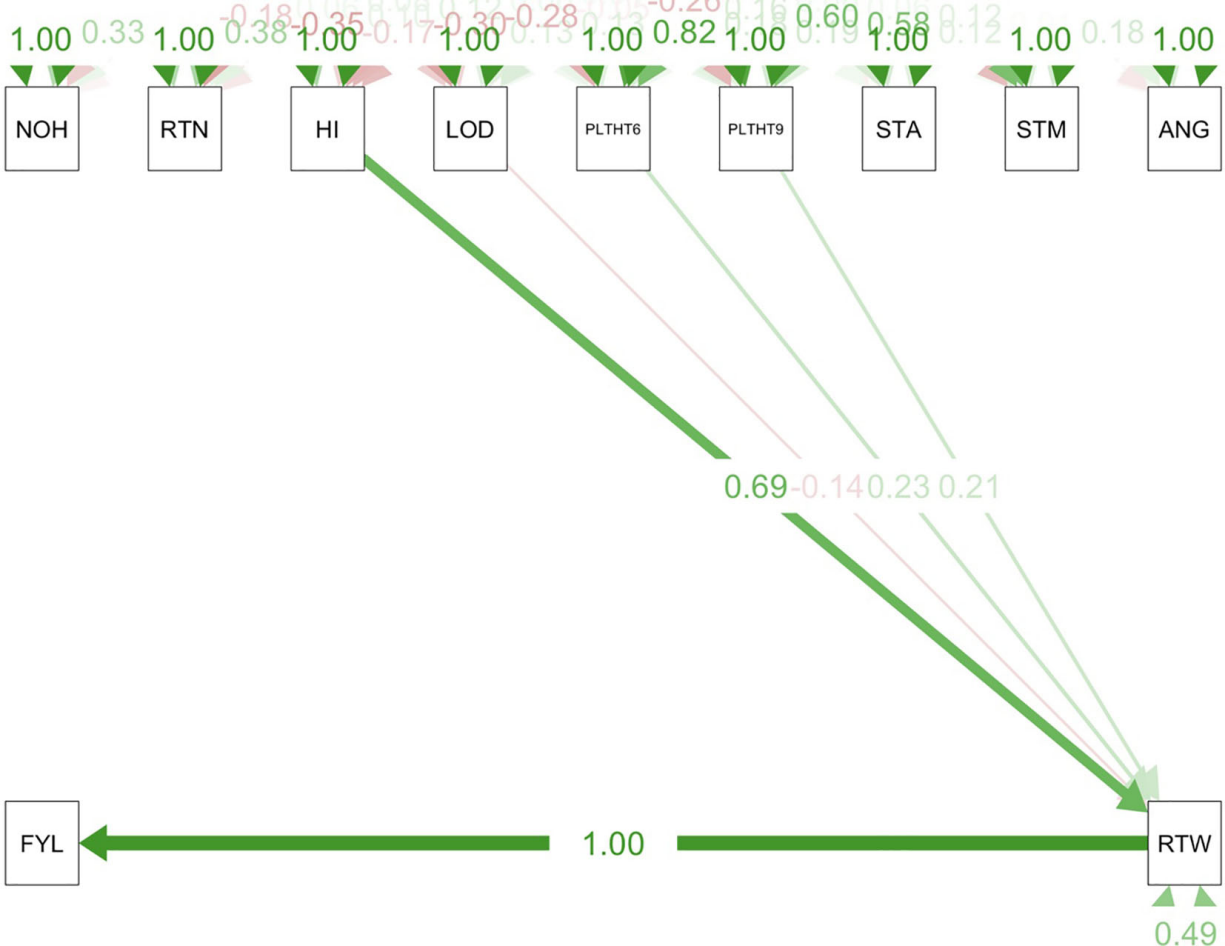
Path coefficient analysis plot of plant architecture and yield traits in Ikenne trial. NOH, number of harvested plants per plot; RTN, number of harvested roots per plot; RTW, root weight; SC, starch content; FYL, fresh root yield; HI, harvest index; LOD, number of lodged plants per plot; PLTHT6, plant height at 6 months after planting; PLTHT9, plant height at 9 months after planting; ANG, angle of branching; STM, stem diameter.

**Figure 10:**
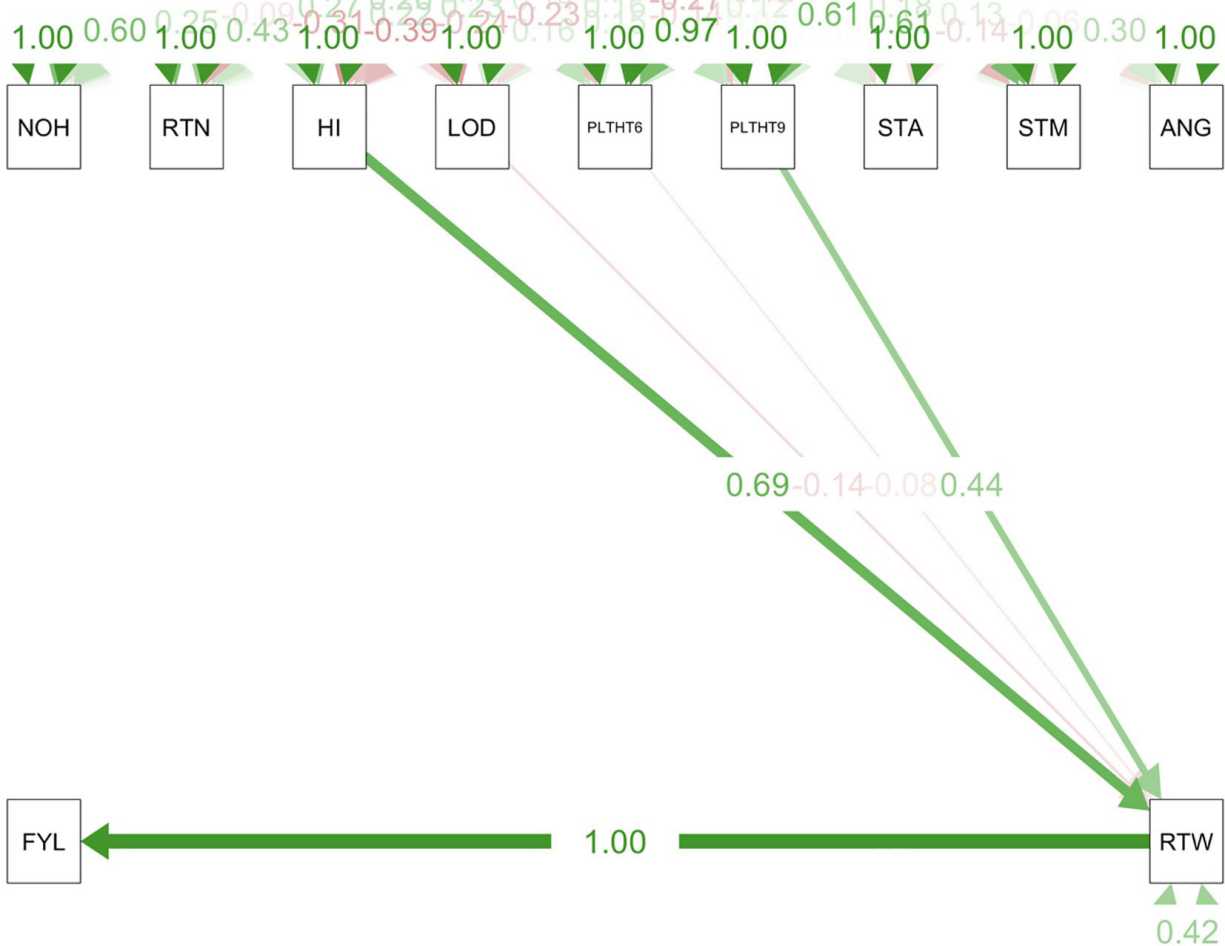
Path coefficient analysis plot of plant architecture and yield traits in Mokwa trial. NOH, number of harvested plants per plot; RTN, number of harvested roots per plot; RTW, root weight; SC, starch content; FYL, fresh root yield; HI, harvest index; LOD, number of lodged plants per plot; PLTHT6, plant height at 6 months after planting; PLTHT9, plant height at 9 months after planting; ANG, angle of branching, and STM, stem diameter.

**Figure 11:**
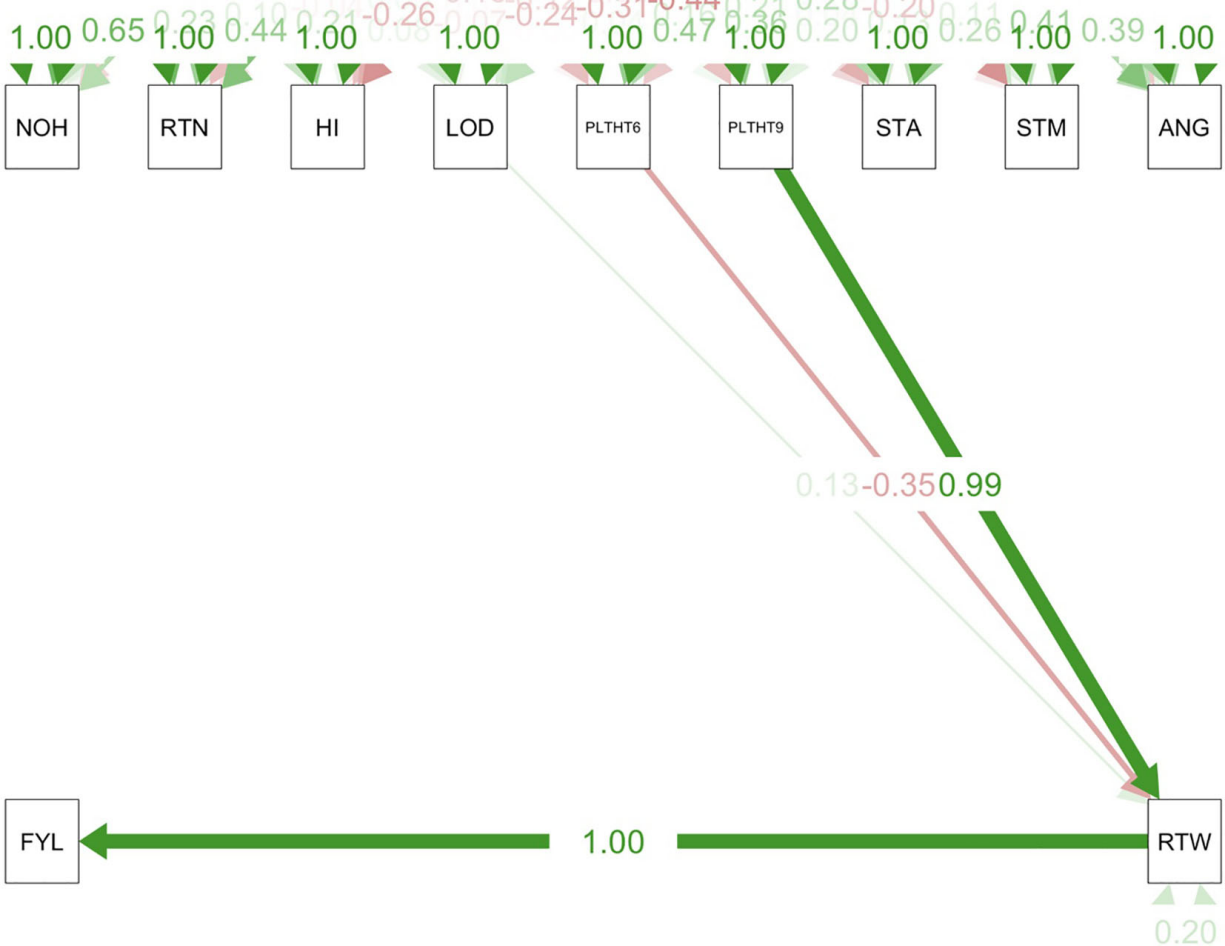
Path coefficient analysis plot of plant architecture and yield traits in Onne trial. NOH, number of harvested plants per plot; RTN, number of harvested roots per plot; RTW, root weight; SC, starch content; FYL, fresh root yield; HI, harvest index; LOD, number of lodged plants per plot; PLTHT6, plant height at 6 months after planting; PLTHT9, plant height at 9 months after planting; ANG, angle of branching; STM, stem diameter.

Path coefficient analysis demonstrated that plant architectural traits had both direct and indirect effects on fresh root yield and fresh root weight in cassava. For the trials conducted in all three test environments (**Figures 9**–**11**), RTWT had an absolute (1.0) positive contribution to FYLD. However, HI had a strong positive contribution to RTWT (0.69) in the trials conducted at Ikenne and Mokwa (**Figures 9**, **10**), whereas it had no contribution to the output of this trait in the trial conducted at Onne. The contributions of PLTHT9 to FYLD through RTWT were also positive across the three test environments, where a near absolute contribution (0.99) was recorded in the trial conducted at Onne (**Figure 11**). In contrast, irregular relationships were observed across the test environments for LODG and PLTHT6 with respect to their contributions to RTWT. For LODG, a negative contribution with a low magnitude (-0.14) was reported in two test environments (**Figures 9**, **10**), whereas the contribution was positive (0.13) in the third test environment (**Figure 11**). Similar results were obtained for PLTHT6, which had a positive contribution (0.23) to RTWT in the Ikenne trial (**Figure 9**), but negative effects on this trait in the Mokwa (-0.08) and Onne (-0.35) trials (**Figures 10**, **11**).

### Marker-trait analysis

**Figure 12:**
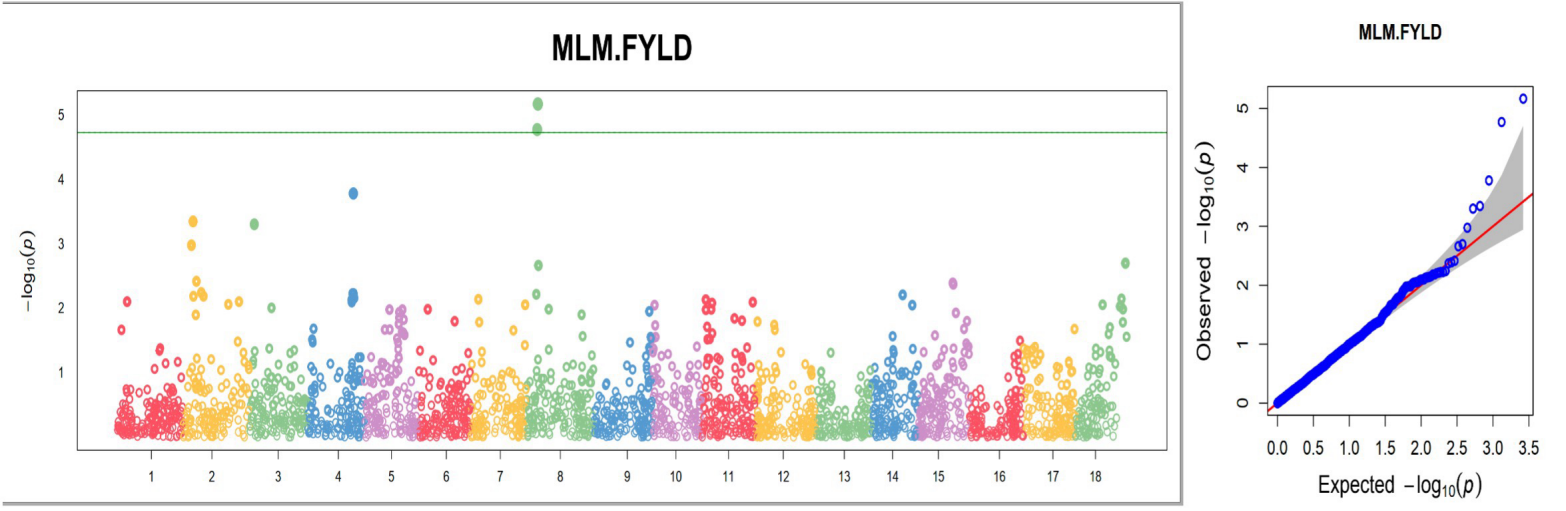
Manhattan and quantile–quantile (Q-Q) plots of significant MTAs for one of the nine plant architecture and yield-related traits (FYLD) from the GWAS analysis conducted on 420 cassava accessions and 2,527 SNP markers.

Four hundred and twenty (420) accessions, 2,527 DArTag, and 54,574 DArTSeq SNP markers were used in GWAS analyses. The GWAS conducted using the DArTag markers and the mixed linear model revealed 16 significant marker-trait hits for eight plant architecture traits (ANGBR9, BRNHB9, PLTHT6, PLTHT9, BRNLEV3, BRNLEV9, SHTWT, and TYLD) and three yield traits (RTWT, FYLD, and DYLD) (**Table 2**; **Figure 12**). Some of the significant SNP hits were location specific while some were detected across all the locations (**Table 3**). Significant SNPs were found on chromosomes 1, 2, 6, 8, 11, 12, 14, and 15. ANGBR9 had significant SNPs on chromosomes 12 and 14, while three chromosomes had significant marker-trait associations (MTAs) for more than one trait: chromosome 8 for PLTHT9, FYLD, and RTWT; chromosome 6 for SHTWT and TYLD; and chromosome 12 for ANGBR9 and BRNHB9. The remaining traits, DYLD, BRNLEV3, BRNLEV9, and BRNHB9, only had significant MTAs on chromosomes 2, 1, 15, and 12, respectively (**Table 2**; **Figure 12**). Further analyses of the 16 significant SNP hits led to the identification of 21 putative candidate genes for nine traits (**Table 4**). These included ANGBR9 (7), PLTHT9 (2), BRNLEV3 (1), BRNLEV9 (6), FYLD (1), RTWT (1), SHTWT (1), TYLD (1), and DYLD (3) (**Table 4**).

**Table 2:** Summary of top significant SNPs for plant architecture and yield traits of cassava.

Trait	SNP_ID	Chromosome number	Position	Value of P	Minor allele frequency	Phenotypic variance explained
ANGBR9	CassV7_chr12_436985	12	436985	2.37E^-06^	0.0782	10.01
	CassV7_chr12_674448	12	674448	1.43E^-06^	0.0687	4.62
	CassV7_chr14_18810275	14	18810275	1.65E^-05^	0.3949	7.53
PLTHT6	CassV7_chr08_17260363	8	17260363	9.89E^-06^	0.4866	21.01
PLTHT9	CassV7_chr08_17260363	8	17260363	8.56E^-06^	0.4866	19.57
FYLD	CassV7_chr08_6710616	8	6710616	1.70E^-05^	0.3165	0
	CassV7_chr08_7101298	8	7101298	6.80E^-06^	0.2918	12.7
BRNLEV3	CassV7_chr15_15530801	15	15530801	1.40E^-05^	0.491	16.18
BRNLEV9	CassV7_chr01_27643909	1	27643909	1.06E^-05^	0.2737	0
	CassV7_chr01_28103607	1	28103607	5.42E^-06^	0.2737	0
	CassV7_chr01_28262168	1	28262168	6.50E^-06^	0.3431	5.06
	CassV7_chr01_29155618	1	29155618	9.29E^-06^	0.4982	9.22
	CassV7_chr01_29944386	1	29944386	9.97E^-07^	0.3303	0
	CassV7_chr01_30312867	1	30312867	9.97E^-07^	0.3303	10.54
DYLD	CassV7_chr02_6125643	2	6125643	1.76E^-05^	0.3859	12.57
RTWT	CassV7_chr08_6710616	8	6710616	1.70E^-05^	0.3165	0
	CassV7_chr08_7101298	8	7101298	6.80E^-06^	0.2918	12.7
BRNHB9	CassV7_chr12_674448	12	674448	1.14E^-05^	0.0691	21.72
SHTWT	CassV7_chr06_25850871	6	25850871	7.15E^-06^	0.1906	23.81
TYLD	CassV7_chr06_25850871	6	25850871	7.15E^-06^	0.1906	23.81

**Table 3:** Matrix of traits by environments of significant SNPs for plant architecture and yield traits of cassava across the test environments.

Traits/environments	Ikenne	Mokwa	Onne	MET
Level of Branching at 3 Months after Planting		⋄		
Level of Branching at 9 Months after Planting	⋄		⋄	
Fresh Root Yield			⋄	
Fresh Root Weight			⋄	
Shoot Weight			⋄	
Top Yield			⋄	
Dry Yield		⋄	⋄	
Plant Height at 6 Months after Planting	⋄			⋄
Plant Height at 9 Months after Planting	⋄			⋄
Angle of Branching		⋄		
Branching Habit				⋄

MET, Multi-Environment Trial.

**Table 4:** Gene annotation of the significant SNPs for plant architecture and yield traits.

Trait	SNP_ID	Chromosome number	Gene ID	Gene annotation
PLTHT9	CassV7_chr08_17260363	8	Manes.08G075800	Oligopeptide transporter 1-like
	CassV7_chr08_17260363	8	Manes.08G075650	Uncharacterized protein
ANGBR9	CassV7_chr12_436985	12	Manes.12G003700	Thaumatin-like protein
	CassV7_chr12_436985	12	Manes.12G003900	Legume lectin domain-containing protein
	CassV7_chr12_436985	12	Manes.12G003600	Protein TIC 20
	CassV7_chr12_436985	12	Manes.12G003800	Thaumatin-like protein
	CassV7_chr12_674448	12	Manes.12G007000	Glutaredoxin domain-containing protein
	CassV7_chr12_674448	12	Manes.12G007100	Uncharacterized protein
	CassV7_chr12_674448	12	Manes.12G006900	Exocyst subunit Exo70 family protein
	CassV7_chr14_18810275	14	NA	
BRNLEV3	CassV7_chr15_15530801	15	Manes.15G171966	Uncharacterized protein
BRNLEV9	CassV7_chr01_27643909	1	Manes.01G122000	Zinc finger ZPR1-type domain-containing protein
	CassV7_chr01_28103607	1	Manes.01G128600	BED-type domain-containing protein
	CassV7_chr01_28262168	1	Manes.01G130800	PH domain-containing protein
	CassV7_chr01_29155618	1	Manes.01G142400	Uncharacterized protein
	CassV7_chr01_29944386	1	Manes.01G154200	K04523 - ubiquilin (UBQLN, DSK2)
	CassV7_chr01_30312867	1	Manes.01G159600	SMP-LTD domain-containing protein
FYLD	CassV7_chr08_7101298	8	Manes.08G058000	Uncharacterized protein
	CassV7_chr08_6710616	8	NA	
RTWT	CassV7_chr08_7101298	8	Manes.08G058000	Uncharacterized protein
	CassV7_chr08_6710616	8	NA	
SHTWT	CassV7_chr06_25850871	6	Manes.06G122900	Membrane-anchored ubiquitin-fold protein
DYLD	CassV7_chr02_6125643	2	Manes.02G069600	DUF4005 domain-containing protein
	CassV7_chr02_6125643	2	Manes.02G069700	Uncharacterized protein MANES_02G069700
	CassV7_chr02_6125643	2	Manes.02G069800	C2H2-type domain-containing protein
TYLD	CassV7_chr06_25850871	6	Manes.06G122900	Membrane-anchored ubiquitin-fold protein

**Figure 13:**
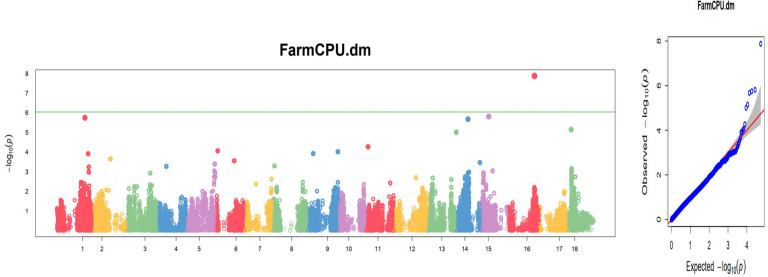
Manhattan and quantile–quantile (Q-Q) plots of significant MTAs for one of the nine plant architecture and yield-related traits (DM) from the GWAS analysis conducted on 420 cassava accessions and 54,574 SNP markers.

Only eight traits had significant SNP hits when DArTseq markers and the FarmCPU model were used (**Table 5**; **Figure 13**). These included six plant architecture traits (ANGBR9, PT, VR, BRNLEV6, BRNLEV9, and PPSTD9) and two yield traits (HI and DM). The 17 significant SNPs recorded for these traits were found on chromosomes 1, 3, 4, 5, 6, 8, 9, 11, 12, 15, and 16. A total of 19 putative candidate genes were identified near the significant SNPs (**Table 6**). Significant SNP were validated using MLMe analysis. The validation procedure confirmed the genuineness of each significant DArTag marker as a result of the mixed linear model performed in GAPIT, and the validation analyses performed in GCTA were identical. In contrast, only one of the significant SNPs on chromosome 6 detected using DArTSeq markers and the FarmCPU model (S6_21336567) was detected by the MLMe analysis conducted in GCTA. The Q-Q plots revealed a huge deflection from the observed to expected variation when DArTSeq markers and the FarmCPU model were used, whereas little or no deflection was observed when using DArTag markers and the mixed linear model. This suggests that both the SNPs and candidate genes discovered through the GWAS conducted using the DArTSeq markers and the FarmCPU model were probably artifacts.

**Table 5:** Summary of top significant SNPs for plant architecture and yield traits of cassava.

Trait	SNP_ID	Chromosome number	Position	Value of P	Minor allele frequency	Phenotypic variance explained
ANGBR9	S1_19494690	1	19494690	6.77E^-07^	0.15944	0.85688
	S1_23883230	1	23883230	4.58E^-07^	0.19515	18.5628
PLTTP	S3_1388806	3	1388806	1.17E^-07^	0.46495	0
	S5_10702830	5	10702830	1.80E^-08^	0.44393	3.88491
	S15_10073295	15	10073295	2.78E^-07^	0.1729	1.60299
VR	S4_5944464	4	5944464	2.94E^-08^	0.42523	1.12237
	S16_2295914	16	2295914	2.73E^-08^	0.30958	1.87443
	S17_17302312	17	17302312	5.68E^-08^	0.43107	5.10115
BRNLEV6	S8_27519983	8	27519983	5.53E^-07^	0.15278	18.6092
	S11_16097795	11	16097795	5.67E^-08^	0.10301	15.8714
BRNLEV9	S5_22148410	5	22148410	1.20E^-09^	0.45718	0.61819
	S11_16097795	11	16097795	3.30E^-08^	0.10301	22.6755
DM	S16_24715889	16	24715889	1.33E^-08^	0.14419	24.3844
HI	S9_3493565	9	3493565	1.32E^-13^	0.31481	5.37937
	S12_29499402	12	29499402	1.81E^-07^	0.4456	5.60214
	S16_22907596	16	22907596	1.43E^-08^	0.06134	7.05957
NOHAV	S6_21336567	6	21336567	9.00E^-07^	0.08912	32.7769

**Table 6:** Gene annotation of the significant SNPs for plant architecture and yield traits.

Trait	Marker	Position	Chromosome	Gene ID	Gene annotation
ANGBR9	S1_19494690	19494690	1	Manes.01G056000	glucan endo-1,3-beta-D-glucosidase
				Manes.01G056100	Ubiquitin carboxyl-terminal hydrolase
	S1_23883230	23883230	1	Manes.01G077800	Nitrate-transporting ATPase
				Manes.01G077900	Uncharacterized Manes.01G077900
PLTTP	S3_1388806	1388806	3	Manes.03G016100	DYW domain-containing protein
				Manes.03G016200	AAA+ ATPase domain-containing protein
				Manes.03G016300	Adenine Nucleotide Transporter
	S15_10073295	10073295	15	Manes.15G122400	9-cis-epoxycarotenoid dioxygenase
VR	S4_5944464	5944464	4	Manes.04G041500	RBR-type E3 ubiquitin transferase
	S16_2295914	2295914	16	Manes.16G020700	F-box domain-containing protein
BRNLEV6	S11_16097795	16097795	11	Manes.11G089900	Triosephosphate isomerase
BRNLEV9	S5_22148410	22148410	5	Manes.05G148900	Cytokinin riboside 5’-monophosphate phosphoribohydrolase
				Manes.05G148800	Endoglucanase
				Manes.05G148700	Myb/SANT-like domain-containing protein
	S11_16097795	16097795	11	Manes.11G089900	Triosephosphate isomerase
DM	S16_24715889	24715889	16	NA	NA
HI	S9_3493565	3493565	9	Manes.09G018900	Glycosyltransferase
	S12_29499402	29499402	12	Manes.12G109600	RING-type E3 ubiquitin transferase
	S16_22907596	22907596	16	Manes.16G062600	JmjC domain-containing protein
NOHAV	S6_21336567	21336567	6	Manes.06G073700	TauD/TfdA-like domain-containing protein

## Discussion

Plant height and stem diameter are primary plant architectural traits that significantly influence the position and alignment of other plant architectural traits (Laurans et al., 2024). The negative correlation between FYLD, the primary predictor of yield, and PLTH is consistent with the conclusions of (Edet et al., 2015), who also reported a negative relationship between the two traits. This implies that sustainable improvement in FYLD productivity in cassava could be achieved by attaining a balance in the expression of these inversely related traits.

Heritability is an essential parameter in plant breeding, as it indicates the potential for the genetic improvement of a trait through selection. A couple of plant architecture and yield-related traits were reported to have moderately high to high broad-sense heritability, including FYLD (0.67), DM (0.68), HI (0.86), BRNHT6 (0.86), BRNHT9 (0.88), PLTHT9 (0.80), and SC (0.70). Similar results were reported (Santos et al., 2023) for FYLD and for DM. High SC content is one of the selling points of cassava varieties targeted for industrial use. The high value of broad-sense heritability recorded for SC in this study corroborated the findings of (Namakula and Nuwamanya, 2022), who reported a heritability value of 0.76. Given that PLTHT9, PLTHT6, STMDI9, HI, FYLD, SC, and LODG have high values of broad sense heritability (0.80), (0.83), (0.73), (0.86), (0.67), (0.7), and (0.69), respectively, there is significant potential for genetic improvement of these traits in cassava through selection, thereby making it feasible to modify these traits to develop cassava varieties with optimal architecture and increased yield.

### Marker coverage and SNP density

The DArTseq marker system uses a genome complexity reduction approach, whereby restriction enzymes randomly cut at regions with high frequencies of cutting sites, resulting in an uneven SNP distribution across the genome (Kilian et al., 2012).

This results in the omission and underrepresentation of certain genomic regions, such as repetitive sequences, heterochromatic regions, and areas lacking restriction enzyme recognition sites, leading to observed gaps in genome coverage (Wenzl et al., 2004; Kilian et al., 2012; Sánchez-Sevilla et al., 2015). In contrast, DArTag employs a targeted genotyping approach, where pre-selected SNPs are evenly distributed across the genome, ensuring uniform coverage and minimizing gaps (Hardenbol et al., 2003; Hardigan et al., 2023). Large gaps, as observed with DArTseq markers, have a significant impact on GWAS results. Regions with sparse SNP coverage may not capture causal variants or nearby loci in linkage disequilibrium (LD), leading to missed associations with traits (Kilian et al., 2012; Sánchez-Sevilla et al., 2015).

### Marker-trait association analysis

Subsequent to the output of the significant SNPs validation, the discussion on the GWAS would be limited to the results obtained through the GWAS conducted using the DArTag markers and the mixed linear model. GWAS is a crucial method for investigating the genetic underlining of complex plant traits (Zhu et al., 2008; Bentley et al., 2022; Susmitha et al., 2023). Of the 11 traits with significant SNPs, only ANGBR9 had significant SNPs on multiple chromosomes (chromosomes 12 and 14). This suggests that this trait is complex and influenced by multiple genes. A similar result was recorded for the angle of branching in a GWAS experiment conducted on cotton across three test environments (Shao et al., 2022) and for fresh root yield in cassava (Hohenfeld et al., 2022).

We found evidence of pleiotropy with some significant SNPs associated with multiple traits (e.g., CassV7_chr12_674448), which were recorded on chromosome 12 for both ANGBR9 and BRNHB9. This implies that the genes controlling these traits could co-segregate and be inherited together (Abah et al., 2024). reported a similar outcome for cassava, where a significant SNP (S4_8840623) was associated with the productivity of dry yield and bulking index. He also reported that these SNPs (S10_2319500, S2_1937678, and S3_3324735) independently influenced both the starch and dry matter contents.

Similarly, significant MTAs for PLTHT9, FYLD, and RTWT were found on chromosome 8, validating the results of the path coefficient analysis, where PLTHT9 had a significant positive contribution to FYLD through RTWT across the test environments with an almost absolute contribution (0.99) to FYLD in the trial at Onne (**Figure 11**) (Rabbi et al., 2017). also reported the colocalization of SNPs that control chromameter value, colour chart, and dry matter content in fresh cassava roots, while (Villwock et al., 2025) reported similar results for total carotenoid and dry matter contents.

Plant height is an essential component of plant architecture that influences crop productivity through its effects on planting density, amount of insolation received, and resistance to lodging (Fei et al., 2022). The efficiency of mechanical harvesting can be influenced by plant height (Yan et al., 2019). The crux of this research is based on identifying genomic regions that could be exploited to develop cassava varieties with ideal plant architecture and improved yield. The identification of these important genomic regions on chromosome 8 that control plant height (CassV7_chr08_17260363) and fresh root yield (CassV7_chr08_6710616 and CassV7_chr08_7101298) constitutes a huge leap towards the realization of the core objective of this research, aimed at unraveling the genetics of plant architecture and its effects on yield in cassava, as well as suitability for mechanized farming.

### Putative candidate genes linked to marker loci for traits associated with plant architecture and yield

Of the 21 putative candidate genes identified, six were found to promote yield, plant growth, and development. These include two genes identified on the angle of branching: Manes.12G003900, which encodes a legume lectin domain-containing protein (Aleksander et al., 2023) that promotes plant growth and development and enhances plant defence mechanisms against pathogens (Katoch and Tripathi, 2021), and Manes.12G007000, which encodes a glutaredoxin domain-containing protein (Aleksander et al., 2023) that regulates gene expression and signal transduction and contributes to plant growth and development (Rouhier et al., 2006). Similarly, for plant height, we identified Manes.08G075800, which encodes an oligopeptide transporter 1-like protein (Aleksander et al., 2023). This gene is involved in plant development and adaptation to stress (Liu et al., 2012; Zhai et al., 2014). The level of branching, a measure of flowering events that the plant has undergone, is a fundamental aspect of plant architecture that influences the plant’s ability to capture light, distribute nutrients, and overall biomass production and yield potential (Pineda et al., 2020). The candidate gene associated with the level of branching, Manes.01G130800, encodes a PH (Pleckstrin Homology) domain-containing protein (Aleksander et al., 2023), which influences root development in plants through its interactions with phosphoinositides and phosphatidic acid (Lemmon, 2010). Similar to the study by (Baguma et al., 2024), on chromosome 15, we identified a genomic region linked with this trait that was approximately 3,783,500 bp apart from the one reported by (Baguma et al., 2024). The candidate gene Manes.15G171966, identified in this region in this study, is a novel candidate gene, while those reported by (Baguma et al., 2024) have well-documented functions. This novel candidate gene presents a prospect for the validation of genes involved in plant architecture and flowering in cassava breeding. Yield improvement is a critical component of all crop improvement programs. Plant breeders are continually developing new strategies and technologies to enhance crop productivity and ensure global food security. Fresh root yield and root weight are important metrics for evaluating cassava yield. The candidate gene Manes.08G058000 identified in FYLD and RTWT encodes an MYB-like (myeloblastosis) DNA-binding protein (Goodstein et al., 2014). MYB is a transcription factor that plays a crucial role in regulating various cellular processes in plants, including the cell cycle and cell morphogenesis (Ambawat et al., 2013), biotic and abiotic stress responses (Roy, 2016), secondary metabolism, such as anthocyanin biosynthesis (Roy, 2016), and plant development (Katiyar et al., 2012; Roy, 2016). Similarly, Manes.02G069600, a candidate gene identified in DYLD, encodes a C2H2-type domain-containing protein (Aleksander et al., 2023). C2H2-type domain-containing proteins affect the metabolic pathways involved in photosynthetic processes (Lakshmanan et al., 2014; Habibpourmehraban et al., 2022).

The discovered novel candidate genes Manes.12G007100 (ANGBR9), Manes.08G075650 (PLTHT9), Manes.08G058000 (FYLD and RTWT), Manes.02G069700 (DYLD), Manes.01G142400, and Manes.15G171966 (BRNLEV3 and BRNLEV9) provide insights into the genetic pathways through which these traits could be improved.

## Conclusion

The genetic underpinnings of plant architecture and yield-associated traits in cassava were examined in this study through a genome-wide association study (GWAS). This investigation involved 420 cassava accessions and employed two genotyping platforms, DArTseq and DArTag, across three distinct environments. Sixteen significant, validated marker-trait associations were discovered using the DArTag markers, compared to only one reported using the DarTseq markers. This highlights the importance of maintaining a balance between the density and distribution of markers for more reliable GWAS results.

The significant marker-trait associations were linked to important genomic regions that could enhance marker-assisted selection for suitable plant architecture and increased yield in cassava breeding. These include putative candidate genes for angle of branching (7), plant height (2), level of branching (7), fresh root yield and weight (1), fresh shoot weight and top yield (1), and dry yield (3). These candidate genes exhibit various functions related to plant architecture, adaptation, yield (root development), plant growth, and stress response. Out of the 21 putative candidate genes identified in this study, six novel genes (Manes.08G075650, Manes.12G007100, Manes.15G171966, Manes.01G142400, Manes.08G058000, Manes.02G069700) were discovered. This represents a significant contribution to the existing knowledge. These findings provide a gateway for exploring the genetic control of cassava plant architecture and yield. This research output will provide cassava breeders with the genetic and molecular leverage required to fast-track cassava improvement in terms of yield, productivity, and adaptation for mechanized cultivation and industrial use.

## Data Availability

The raw data supporting the conclusions of this article will be made available by the authors, without undue reservation.
