# Supplementary material for: Genome-wide association study identifies novel genes for plant architecture and yield traits in cassava (Manihot esculenta Crantz)
Source: Front Plant Sci. 2025 Sep 10;16:1660789. doi: 10.3389/fpls.2025.1660789 (PMC12457381; doi:10.3389/fpls.2025.1660789)
Supplement: Supplementary file 1 [file Table1.docx]

**Table S1: Details of the Cassava Accessions used for the experiment.**

| Accession ID | Pedigree | Entry Type |
| --- | --- | --- |
| IITA-TMS-IBA000070 | TMEB459/? | Check |
| IITA-TMS-IBA30572 | 58308/BRANCA DE SANTA CATARINA | Check |
| IITA-TMS-IBA980581 | IITA-TMS-IBA980581 | Check |
| IITA-TMS-IBA982101 | IITA-TMS-IBA951181/71173 | Check |
| NR130124 | NR130124 | Check |
| TMEB419 | TMEB419 | Check |
| TMS13F1160P0004 | IITA-TMS-IBA993073/IITA-TMS-IBA051740 | Test |
| TMS13F1307P0016 | IITA-TMS-IBA030060/IITA-TMS-IBA010903 | Test |
| TMS13F1343P0022 | IITA-TMS-IBA970425/IITA-TMS-IBA930007 | Test |
| TMS13F1343P0044 | IITA-TMS-IBA970425/IITA-TMS-IBA930007 | Test |
| TMS18F1001P0014 | TMS15F1004P0001/TMS15F1159P0006 | Test |
| TMS18F1001P0020 | TMS15F1004P0001/TMS15F1159P0006 | Test |
| TMS18F1003P0003 | TMS15F1021P0002/TMS15F1132P0003 | Test |
| TMS18F1003P0009 | TMS15F1021P0002/TMS15F1132P0003 | Test |
| TMS18F1003P0014 | TMS15F1021P0002/TMS15F1132P0003 | Test |
| TMS18F1004P0008 | TMS15F1021P0002/TMS15F1153P0009 | Test |
| TMS18F1005P0023 | TMS15F1021P0002/TMS15F1156P0014 | Test |
| TMS18F1007P0004 | TMS15F1021P0002/TMS15F1310P0019 | Test |
| TMS18F1007P0005 | TMS15F1021P0002/TMS15F1310P0019 | Test |
| TMS18F1010P0011 | TMS15F1025P0001/TMS15F1305P0021 | Test |
| TMS18F1016P0010 | TMS15F1069P0007/TMS15F1398P0008 | Test |
| TMS18F1018P0001 | TMS15F1072P0005/TMS15F1367P0001 | Test |
| TMS18F1019P0018 | TMS15F1072P0005/TMS15F1398P0008 | Test |
| TMS18F1020P0027 | TMS15F1072P0006/TMS15F1326P0004 | Test |
| TMS18F1024P0020 | TMS15F1072P0012/TMS15F1398P0008 | Test |
| TMS18F1025P0041 | TMS15F1072P0031/TMS15F1156P0014 | Test |
| TMS18F1026P0009 | TMS15F1072P0031/TMS15F1196P0010 | Test |
| TMS18F1028P0003 | TMS15F1073P0005/TMS15F1153P0009 | Test |
| TMS18F1028P0030 | TMS15F1073P0005/TMS15F1153P0009 | Test |
| TMS18F1038P0010 | TMS15F1080P0004/TMS15F1153P0009 | Test |
| TMS18F1038P0056 | TMS15F1080P0004/TMS15F1153P0009 | Test |
| TMS18F1038P0087 | TMS15F1080P0004/TMS15F1153P0009 | Test |
| TMS18F1040P0032 | TMS15F1080P0004/TMS15F1318P0024 | Test |
| TMS18F1040P0044 | TMS15F1080P0004/TMS15F1318P0024 | Test |
| TMS18F1040P0054 | TMS15F1080P0004/TMS15F1318P0024 | Test |
| TMS18F1041P0002 | TMS15F1080P0007/TMS15F1305P0017 | Test |
| TMS18F1041P0005 | TMS15F1080P0007/TMS15F1305P0017 | Test |
| TMS18F1042P0009 | TMS15F1080P0007/TMS15F1367P0001 | Test |

| Accession ID | Pedigree | Entry Type |
| --- | --- | --- |
| TMS18F1044P0013 | TMS15F1080P0012/TMS15F1159P0006 | Test |
| TMS18F1047p0012 | TMS15F1080P0012/TMS15F1398P0008 | Test |
| TMS18F1047P0045 | TMS15F1080P0012/TMS15F1398P0008 | Test |
| TMS18F1047P0046 | TMS15F1080P0012/TMS15F1398P0008 | Test |
| TMS18F1047P0049 | TMS15F1080P0012/TMS15F1398P0008 | Test |
| TMS18F1048P0023 | TMS15F1081P0003/TMS15F1072P0018 | Test |
| TMS18F1051P0059 | TMS15F1081P0003/TMS15F1156P0001 | Test |
| TMS18F1051P0063 | TMS15F1081P0003/TMS15F1156P0001 | Test |
| TMS18F1055P0036 | TMS15F1081P0003/TMS15F1367P0001 | Test |
| TMS18F1055P0044 | TMS15F1081P0003/TMS15F1367P0001 | Test |
| TMS18F1056P0016 | TMS15F1081P0003/TMS15F1398P0008 | Test |
| TMS18F1056P0019 | TMS15F1081P0003/TMS15F1398P0008 | Test |
| TMS18F1056P0066 | TMS15F1081P0003/TMS15F1398P0008 | Test |
| TMS18F1058P0019 | TMS15F1100P0005/TMS15F1132P0013 | Test |
| TMS18F1058P0020 | TMS15F1100P0005/TMS15F1132P0013 | Test |
| TMS18F1059P0015 | TMS15F1100P0005/TMS15F1153P0009 | Test |
| TMS18F1060P0067 | TMS15F1100P0005/TMS15F1156P0014 | Test |
| TMS18F1063P0008 | TMS15F1103P0006/TMS15F1153P0009 | Test |
| TMS18F1064P0027 | TMS15F1103P0006/TMS15F1156P0014 | Test |
| TMS18F1065P0037 | TMS15F1103P0006/TMS15F1195P0009 | Test |
| TMS18F1068P0063 | TMS15F1103P0006/TMS15F1305P0007 | Test |
| TMS18F1068P0077 | TMS15F1103P0006/TMS15F1305P0007 | Test |
| TMS18F1069P0023 | TMS15F1103P0006/TMS15F1318P0009 | Test |
| TMS18F1069P0033 | TMS15F1103P0006/TMS15F1318P0009 | Test |
| TMS18F1073P0035 | TMS15F1104P0001/TMS15F1156P0014 | Test |
| TMS18F1073P0038 | TMS15F1104P0001/TMS15F1156P0014 | Test |
| TMS18F1076P0001 | TMS15F1109P0003/TMS15F1156P0014 | Test |
| TMS18F1076P0066 | TMS15F1109P0003/TMS15F1156P0014 | Test |
| TMS18F1076P0077 | TMS15F1109P0003/TMS15F1156P0014 | Test |
| TMS18F1076P0103 | TMS15F1109P0003/TMS15F1156P0014 | Test |
| TMS18F1076P0106 | TMS15F1109P0003/TMS15F1156P0014 | Test |
| TMS18F1076P0112 | TMS15F1109P0003/TMS15F1156P0014 | Test |
| TMS18F1076P0114 | TMS15F1109P0003/TMS15F1156P0014 | Test |
| TMS18F1076P0117 | TMS15F1109P0003/TMS15F1156P0014 | Test |
| TMS18F1076P0121 | TMS15F1109P0003/TMS15F1156P0014 | Test |
| TMS18F1081P0014 | TMS15F1124P0001/TMS15F1214P0002 | Test |
| TMS18F1082P0024 | TMS15F1124P0001/TMS15F1351P0003 | Test |
| TMS18F1083P0028 | TMS15F1124P0001/TMS15F1367P0001 | Test |

| Accession ID | Pedigree | Entry Type |
| --- | --- | --- |
| TMS18F1085P0012 | TMS15F1127P0002/TMS15F1156P0014 | Test |
| TMS18F1090P0031 | TMS15F1130P0012/TMS15F1153P0009 | Test |
| TMS18F1092P0016 | TMS15F1130P0012/TMS15F1396P0004 | Test |
| TMS18F1096P0013 | TMS15F1132P0001/TMS15F1177P0004 | Test |
| TMS18F1096P0024 | TMS15F1132P0001/TMS15F1177P0004 | Test |
| TMS18F1107P0020 | TMS15F1153P0009/TMS15F1177P0004 | Test |
| TMS18F1113P0002 | TMS15F1153P0009/TMS15F1396P0004 | Test |
| TMS18F1113P0038 | TMS15F1153P0009/TMS15F1396P0004 | Test |
| TMS18F1114P0039 | TMS15F1153P0009/TMS15F1397P0001 | Test |
| TMS18F1120P0004 | TMS15F1154P0002/TMS15F1196P0010 | Test |
| TMS18F1120P0006 | TMS15F1154P0002/TMS15F1196P0010 | Test |
| TMS18F1121P0029 | TMS15F1154P0002/TMS15F1318P0024 | Test |
| TMS18F1127P0011 | TMS15F1154P0015/TMS15F1396P0007 | Test |
| TMS18F1127P0010 | TMS15F1154P0015/TMS15F1396P0007 | Test |
| TMS18F1132P0042 | TMS15F1156P0006/TMS15F1196P0010 | Test |
| TMS18F1132P0078 | TMS15F1156P0006/TMS15F1196P0010 | Test |
| TMS18F1132P0083 | TMS15F1156P0006/TMS15F1196P0010 | Test |
| TMS18F1132P0144 | TMS15F1156P0006/TMS15F1196P0010 | Test |
| TMS18F1134P0010 | TMS15F1156P0014/TMS15F1318P0024 | Test |
| TMS18F1135P0025 | TMS15F1072P0005/TMS15F1326P0004 | Test |
| TMS18F1138P0028 | TMS15F1160P0010/TMS15F1284P0008 | Test |
| TMS18F1138P0030 | TMS15F1160P0010/TMS15F1284P0008 | Test |
| TMS18F1138P0035 | TMS15F1160P0010/TMS15F1284P0008 | Test |
| TMS18F1138P0037 | TMS15F1160P0010/TMS15F1284P0008 | Test |
| TMS18F1139P0076 | TMS15F1160P0010/TMS15F1318P0024 | Test |
| TMS18F1142P0017 | TMS15F1160P0010/TMS15F1396P0004 | Test |
| TMS18F1144P0010 | TMS15F1195P0006/TMS15F1310P0019 | Test |
| TMS18F1149P0024 | TMS15F1196P0010/TMS15F1310P0019 | Test |
| TMS18F1150P0011 | TMS15F1212P0003/TMS15F1132P0001 | Test |
| TMS18F1152P0003 | TMS15F1230P0005/TMS15F1069P0007 | Test |
| TMS18F1153P0053 | TMS15F1230P0005/TMS15F1132P0001 | Test |
| TMS18F1156P0016 | TMS15F1230P0005/TMS15F1300P0002 | Test |
| TMS18F1163P0035 | TMS15F1244P0004/TMS15F1132P0013 | Test |
| TMS18F1163P0042 | TMS15F1244P0004/TMS15F1132P0013 | Test |
| TMS18F1163P0044 | TMS15F1244P0004/TMS15F1132P0013 | Test |
| TMS18F1165P0008 | TMS15F1269P0004/TMS15F1318P0009 | Test |
| TMS18F1165P0018 | TMS15F1269P0004/TMS15F1318P0009 | Test |
| TMS18F1174P0011 | TMS15F1270P0001/TMS15F1328P0001 | Test |

| Accession ID | Pedigree | Entry Type |
| --- | --- | --- |
| TMS18F1175P0053 | TMS15F1276P0003/TMS15F1321P0001 | Test |
| TMS18F1178P0010 | TMS15F1276P0003/TMS15F1159P0001 | Test |
| TMS18F1178P0046 | TMS15F1276P0003/TMS15F1159P0001 | Test |
| TMS18F1179P0008 | TMS15F1276P0003/TMS15F1318P0009 | Test |
| TMS18F1179P0029 | TMS15F1276P0003/TMS15F1318P0009 | Test |
| TMS18F1180P0033 | TMS15F1295P0001/TMS15F1321P0001 | Test |
| TMS18F1191P0002 | TMS15F1305P0021/TMS15F1201P0017 | Test |
| TMS18F1202p0001 | TMS15F1321P0001/TMS15F1024P0006 | Test |
| TMS18F1205P0010 | TMS15F1321P0003/TMS15F1367P0001 | Test |
| TMS18F1207P0008 | TMS15F1324P0004/TMS15F1159P0001 | Test |
| TMS18F1208P0005 | TMS15F1300P0002/TMS15F1072P0012 | Test |
| TMS18F1208P0007 | TMS15F1300P0002/TMS15F1072P0012 | Test |
| TMS18F1209P0015 | TMS15F1326P0004/TMS15F1159P0001 | Test |
| TMS18F1210P0021 | TMS15F1326P0004/TMS15F1367P0001 | Test |
| TMS18F1211P0035 | TMS15F1328P0001/TMS15F1069P0007 | Test |
| TMS18F1215P0027 | TMS15F1333P0001/TMS15F1159P0001 | Test |
| TMS18F1217P0034 | TMS15F1351P0003/TMS15F1069P0007 | Test |
| TMS18F1217P0075 | TMS15F1351P0003/TMS15F1069P0007 | Test |
| TMS18F1217P0091 | TMS15F1351P0003/TMS15F1069P0007 | Test |
| TMS18F1217P0095 | TMS15F1351P0003/TMS15F1069P0007 | Test |
| TMS18F1217P0114 | TMS15F1351P0003/TMS15F1069P0007 | Test |
| TMS18F1217P0121 | TMS15F1351P0003/TMS15F1069P0007 | Test |
| TMS18F1218P0003 | TMS15F1351P0003/TMS15F1072P0031 | Test |
| TMS18F1221P0053 | TMS15F1351P0003/TMS15F1396P0001 | Test |
| TMS18F1222P0012 | TMS15F1367P0001/TMS15F1132P0001 | Test |
| TMS18F1224P0014 | TMS15F1310P0019/TMS15F1156P0014 | Test |
| TMS18F1225P0030 | TMS15F1396P0001/TMS15F1109P0003 | Test |
| TMS18F1231P0011 | TMS15F1403P0005/TMS15F1195P0006 | Test |
| TMS18F1233p0013 | TMS15F1403P0005/TMS15F1321P0003 | Test |
| TMS18F1240P0024 | TMS15F1461P0009/TMS15F1109P0003 | Test |
| TMS18F1240P0026 | TMS15F1461P0009/TMS15F1109P0003 | Test |
| TMS18F1250P0050 | TMS15F1424P0004/TMS15F1195P0006 | Test |
| TMS18F1258P0062 | TMS13F1020P0001/IITA-TMS-IBA930007 | Test |
| TMS18F1258P0147 | TMS13F1020P0001/IITA-TMS-IBA930007 | Test |
| TMS18F1258P0153 | TMS13F1020P0001/IITA-TMS-IBA930007 | Test |
| TMS18F1258P0156 | TMS13F1020P0001/IITA-TMS-IBA930007 | Test |
| TMS18F1259P0131 | TMS13F1020P0001/IITA-TMS-IBA972205 | Test |
| TMS18F1260P0054 | TMS13F1020P0001/TMEB419 | Test |

| Accession ID | Pedigree | Entry Type |
| --- | --- | --- |
| TMS18F1260P0060 | TMS13F1020P0001/TMEB419 | Test |
| TMS18F1261P0005 | TMS13F1020P0001/TMS13F1088P0007 | Test |
| TMS18F1262P0008 | TMS13F1020P0001/TMS13F1122P0005 | Test |
| TMS18F1262P0015 | TMS13F1020P0001/TMS13F1122P0005 | Test |
| TMS18F1263P0037 | TMS13F1053P0010/IITA-TMS-IBA000070 | Test |
| TMS18F1264P0058 | TMS13F1053P0010/IITA-TMS-IBA030060A | Test |
| TMS18F1265P0030 | TMS13F1053P0010/IITA-TMS-IBA030060 | Test |
| TMS18F1271P0001 | TMS13F1053P0010/TMS13F1227P0143 | Test |
| TMS18F1272P0037 | TMS13F1053P0010/TMS13F1307P0016 | Test |
| TMS18F1274P0020 | TMS13F1053P0010/TMS13F1343P0022 | Test |
| TMS18F1277P0007 | TMS13F1053P0015/IITA-TMS-IBA930007 | Test |
| TMS18F1277P0031 | TMS13F1053P0015/IITA-TMS-IBA930007 | Test |
| TMS18F1277P0045 | TMS13F1053P0015/IITA-TMS-IBA930007 | Test |
| TMS18F1278P0068 | TMS13F1053P0015/IITA-TMS-IBA972205 | Test |
| TMS18F1278P0098 | TMS13F1053P0015/IITA-TMS-IBA972205 | Test |
| TMS18F1278P0118 | TMS13F1053P0015/IITA-TMS-IBA972205 | Test |
| TMS18F1278P0165 | TMS13F1053P0015/IITA-TMS-IBA972205 | Test |
| TMS18F1278P0183 | TMS13F1053P0015/IITA-TMS-IBA972205 | Test |
| TMS18F1280P0057 | TMS13F1053P0015/TMS13F1088P0007 | Test |
| TMS18F1280P0086 | TMS13F1053P0015/TMS13F1088P0007 | Test |
| TMS18F1280P0091 | TMS13F1053P0015/TMS13F1088P0007 | Test |
| TMS18F1280P0107 | TMS13F1053P0015/TMS13F1088P0007 | Test |
| TMS18F1280P0112 | TMS13F1053P0015/TMS13F1088P0007 | Test |
| TMS18F1280P0126 | TMS13F1053P0015/TMS13F1088P0007 | Test |
| TMS18F1280P0136 | TMS13F1053P0015/TMS13F1088P0007 | Test |
| TMS18F1280P0140 | TMS13F1053P0015/TMS13F1088P0007 | Test |
| TMS18F1280P0151 | TMS13F1053P0015/TMS13F1088P0007 | Test |
| TMS18F1282P0049 | TMS13F1063P0013/IITA-TMS-IBA930007 | Test |
| TMS18F1284P0003 | TMS13F1063P0013/TMEB419 | Test |
| TMS18F1286P0012 | TMS13F1088P0007/IITA-TMS-IBA972205 | Test |
| TMS18F1287P0029 | TMS13F1088P0007/TMS13F1343P0002 | Test |
| TMS18F1287P0039 | TMS13F1088P0007/TMS13F1343P0002 | Test |
| TMS18F1289P0014 | TMS13F1106P0004/TMS13F1088P0007 | Test |
| TMS18F1291P0014 | TMS13F1122P0005/IITA-TMS-IBA972205 | Test |
| TMS18F1291P0026 | TMS13F1122P0005/IITA-TMS-IBA972205 | Test |
| TMS18F1295P0023 | TMS13F1160P0005/TMEB419 | Test |
| TMS18F1295P0034 | TMS13F1160P0005/TMEB419 | Test |
| TMS18F1295P0035 | TMS13F1160P0005/TMEB419 | Test |

| Accession ID | Pedigree | Entry Type |
| --- | --- | --- |
| TMS18F1296P0042 | TMS13F1160P0005/TMS14F1284P0001 | Test |
| TMS18F1296P0071 | TMS13F1160P0005/TMS14F1284P0001 | Test |
| TMS18F1298P0017 | TMS13F1284P0001/IITA-TMS-IBA000070 | Test |
| TMS18F1298P0044 | TMS13F1284P0001/IITA-TMS-IBA000070 | Test |
| TMS18F1299P0005 | TMS13F1284P0001/TMEB419 | Test |
| TMS18F1302P0018 | TMS13F1307P0016/IITA-TMS-IBA000070 | Test |
| TMS18F1303P0027 | TMS13F1307P0016/IITA-TMS-IBA030060 | Test |
| TMS18F1305P0007 | TMS13F1307P0016/IITA-TMS-IBA930007 | Test |
| TMS18F1305P0017 | TMS13F1307P0016/IITA-TMS-IBA930007 | Test |
| TMS18F1305P0019 | TMS13F1307P0016/IITA-TMS-IBA930007 | Test |
| TMS18F1305P0031 | TMS13F1307P0016/IITA-TMS-IBA930007 | Test |
| TMS18F1305P0039 | TMS13F1307P0016/IITA-TMS-IBA930007 | Test |
| TMS18F1305P0040 | TMS13F1307P0016/IITA-TMS-IBA930007 | Test |
| TMS18F1305P0138 | TMS13F1307P0016/IITA-TMS-IBA930007 | Test |
| TMS18F1306P0114 | TMS13F1307P0016/IITA-TMS-IBA972205 | Test |
| TMS18F1306P0127 | TMS13F1307P0016/IITA-TMS-IBA972205 | Test |
| TMS18F1306P0129 | TMS13F1307P0016/IITA-TMS-IBA972205 | Test |
| TMS18F1306P0135 | TMS13F1307P0016/IITA-TMS-IBA972205 | Test |
| TMS18F1306P0149 | TMS13F1307P0016/IITA-TMS-IBA972205 | Test |
| TMS18F1307P0001 | TMS13F1307P0016/TMEB419 | Test |
| TMS18F1308P0077 | TMS13F1307P0016/TMS13F1053P0015 | Test |
| TMS18F1308P0082 | TMS13F1307P0016/TMS13F1053P0015 | Test |
| TMS18F1308P0084 | TMS13F1307P0016/TMS13F1053P0015 | Test |
| TMS18F1308P0114 | TMS13F1307P0016/TMS13F1053P0015 | Test |
| TMS18F1309P0062 | TMS13F1307P0016/TMS13F1069P0024 | Test |
| TMS18F1309P0074 | TMS13F1307P0016/TMS13F1069P0024 | Test |
| TMS18F1310P0088 | TMS13F1307P0016/TMS13F1088P0007 | Test |
| TMS18F1310P0148 | TMS13F1307P0016/TMS13F1088P0007 | Test |
| TMS18F1310P0151 | TMS13F1307P0016/TMS13F1088P0007 | Test |
| TMS18F1310P0168 | TMS13F1307P0016/TMS13F1088P0007 | Test |
| TMS18F1312P0033 | TMS13F1307P0016/TMS13F1227P0143 | Test |
| TMS18F1312P0039 | TMS13F1307P0016/TMS13F1227P0143 | Test |
| TMS18F1313P0001 | TMS13F1307P0016/TMS13F1343P0002 | Test |
| TMS18F1313P0013 | TMS13F1307P0016/TMS13F1343P0002 | Test |
| TMS18F1313P0053 | TMS13F1307P0016/TMS13F1343P0002 | Test |
| TMS18F1314P0007 | TMS13F1307P0016/TMS13F1343P0022 | Test |
| TMS18F1314P0026 | TMS13F1307P0016/TMS13F1343P0022 | Test |
| TMS18F1314P0042 | TMS13F1307P0016/TMS13F1343P0022 | Test |

| Accession ID | Pedigree | Entry Type |
| --- | --- | --- |
| TMS18F1317P0048 | TMS13F1343P0002/IITA-TMS-IBA030060A | Test |
| TMS18F1317P0059 | TMS13F1343P0002/IITA-TMS-IBA030060A | Test |
| TMS18F1317P0119 | TMS13F1343P0002/IITA-TMS-IBA030060A | Test |
| TMS18F1317P0123 | TMS13F1343P0002/IITA-TMS-IBA030060A | Test |
| TMS18F1318P0007 | TMS13F1343P0002/IITA-TMS-IBA930007 | Test |
| TMS18F1318P0016 | TMS13F1343P0002/IITA-TMS-IBA930007 | Test |
| TMS18F1318P0018 | TMS13F1343P0002/IITA-TMS-IBA930007 | Test |
| TMS18F1318P0036 | TMS13F1343P0002/IITA-TMS-IBA930007 | Test |
| TMS18F1318P0054 | TMS13F1343P0002/IITA-TMS-IBA930007 | Test |
| TMS18F1319P0033 | TMS13F1343P0002/IITA-TMS-IBA972205 | Test |
| TMS18F1319P0061 | TMS13F1343P0002/IITA-TMS-IBA972205 | Test |
| TMS18F1319P0100 | TMS13F1343P0002/IITA-TMS-IBA972205 | Test |
| TMS18F1319P0121 | TMS13F1343P0002/IITA-TMS-IBA972205 | Test |
| TMS18F1319P0159 | TMS13F1343P0002/IITA-TMS-IBA972205 | Test |
| TMS18F1320P0032 | TMS13F1343P0002/TMEB419 | Test |
| TMS18F1320P0051 | TMS13F1343P0002/TMEB419 | Test |
| TMS18F1320P0082 | TMS13F1343P0002/TMEB419 | Test |
| TMS18F1320P0086 | TMS13F1343P0002/TMEB419 | Test |
| TMS18F1320P0087 | TMS13F1343P0002/TMEB419 | Test |
| TMS18F1320P0101 | TMS13F1343P0002/TMEB419 | Test |
| TMS18F1322P0019 | TMS13F1343P0002/TMS13F1343P0022 | Test |
| TMS18F1323P0002 | TMS13F1343P0022/IITA-TMS-IBA930007 | Test |
| TMS18F1323P0024 | TMS13F1343P0022/IITA-TMS-IBA930007 | Test |
| TMS18F1323P0041 | TMS13F1343P0022/IITA-TMS-IBA930007 | Test |
| TMS18F1323p0025 | TMS13F1343P0022/IITA-TMS-IBA930007 | Test |
| TMS18F1324P0003 | TMS13F1343P0022/IITA-TMS-IBA972205 | Test |
| TMS18F1324P0046 | TMS13F1343P0022/IITA-TMS-IBA972205 | Test |
| TMS18F1324P0070 | TMS13F1343P0022/IITA-TMS-IBA972205 | Test |
| TMS18F1324P0089 | TMS13F1343P0022/IITA-TMS-IBA972205 | Test |
| TMS18F1324P0092 | TMS13F1343P0022/IITA-TMS-IBA972205 | Test |
| TMS18F1324P0093 | TMS13F1343P0022/IITA-TMS-IBA972205 | Test |
| TMS18F1324P0107 | TMS13F1343P0022/IITA-TMS-IBA972205 | Test |
| TMS18F1324P0171 | TMS13F1343P0022/IITA-TMS-IBA972205 | Test |
| TMS18F1324P0200 | TMS13F1343P0022/IITA-TMS-IBA972205 | Test |
| TMS18F1324p0026 | TMS13F1343P0022/IITA-TMS-IBA972205 | Test |
| TMS18F1325P0032 | TMS13F1343P0022/TMEB419 | Test |
| TMS18F1326P0007 | TMS13F1343P0022/TMS13F1053P0015 | Test |
| TMS18F1327P0006 | TMS13F1343P0022/TMS13F1069P0024 | Test |

| Accession ID | Pedigree | Entry Type |
| --- | --- | --- |
| TMS18F1327P0013 | TMS13F1343P0022/TMS13F1069P0024 | Test |
| TMS18F1328P0027 | TMS13F1343P0022/TMS13F1088P0007 | Test |
| TMS18F1328P0032 | TMS13F1343P0022/TMS13F1088P0007 | Test |
| TMS18F1329P0011 | TMS13F1343P0022/TMS13F1122P0005 | Test |
| TMS18F1330P0008 | TMS13F1343P0038/TMEB419 | Test |
| TMS18F1330P0009 | TMS13F1343P0038/TMEB419 | Test |
| TMS18F1330P0020 | TMS13F1343P0038/TMEB419 | Test |
| TMS18F1331P0013 | TMS13F1362P0004/IITA-TMS-IBA000070 | Test |
| TMS18F1331P0016 | TMS13F1362P0004/IITA-TMS-IBA000070 | Test |
| TMS18F1332P0002 | TMS13F1362P0004/TMEB419 | Test |
| TMS18F1333P0012 | TMS13F1362P0004/TMS13F1343P0038 | Test |
| TMS18F1333p0013 | TMS13F1362P0004/TMS13F1343P0038 | Test |
| TMS18F1334P0003 | TMS13F1377P0018/IITA-TMS-IBA000070 | Test |
| TMS18F1334P0023 | TMS13F1377P0018/IITA-TMS-IBA000070 | Test |
| TMS18F1335P0001 | TMS13F1377P0018/IITA-TMS-IBA030060 | Test |
| TMS18F1335P0021 | TMS13F1377P0018/IITA-TMS-IBA030060 | Test |
| TMS18F1335P0035 | TMS13F1377P0018/IITA-TMS-IBA030060 | Test |
| TMS18F1336P0014 | TMS13F1377P0018/IITA-TMS-IBA030060A | Test |
| TMS18F1336P0015 | TMS13F1377P0018/IITA-TMS-IBA030060A | Test |
| TMS18F1337P0044 | TMS13F1377P0018/IITA-TMS-IBA930007 | Test |
| TMS18F1337P0049 | TMS13F1377P0018/IITA-TMS-IBA930007 | Test |
| TMS18F1338P0013 | TMS13F1377P0018/IITA-TMS-IBA972205 | Test |
| TMS18F1338P0053 | TMS13F1377P0018/IITA-TMS-IBA972205 | Test |
| TMS18F1338P0067 | TMS13F1377P0018/IITA-TMS-IBA972205 | Test |
| TMS18F1338P0085 | TMS13F1377P0018/IITA-TMS-IBA972205 | Test |
| TMS18F1338P0099 | TMS13F1377P0018/IITA-TMS-IBA972205 | Test |
| TMS18F1338P0115 | TMS13F1377P0018/IITA-TMS-IBA972205 | Test |
| TMS18F1338P0116 | TMS13F1377P0018/IITA-TMS-IBA972205 | Test |
| TMS18F1339P0012 | TMS13F1377P0018/TMEB419 | Test |
| TMS18F1339P0015 | TMS13F1377P0018/TMEB419 | Test |
| TMS18F1340P0001 | TMS13F1377P0018/TMS13F1069P0024 | Test |
| TMS18F1340P0030 | TMS13F1377P0018/TMS13F1069P0024 | Test |
| TMS18F1341P0037 | TMS13F1377P0018/TMS13F1122P0005 | Test |
| TMS18F1341P0044 | TMS13F1377P0018/TMS13F1122P0005 | Test |
| TMS18F1342P0003 | TMS13F1377P0018/TMS13F1343P0002 | Test |
| TMS18F1342P0017 | TMS13F1377P0018/TMS13F1343P0002 | Test |
| TMS18F1343P0012 | TMS13F1053P0010/IITA-TMS-IBA930007 | Test |
| TMS18F1343P0014 | TMS13F1053P0010/IITA-TMS-IBA930007 | Test |

| Accession ID | Pedigree | Entry Type |
| --- | --- | --- |
| TMS18F1343P0064 | TMS13F1053P0010/IITA-TMS-IBA930007 | Test |
| TMS18F1343P0153 | TMS13F1053P0010/IITA-TMS-IBA930007 | Test |
| TMS18F1343P0171 | TMS13F1053P0010/IITA-TMS-IBA930007 | Test |
| TMS18F1344P0003 | TMS13F1053P0010/IITA-TMS-IBA972205 | Test |
| TMS18F1344P0014 | TMS13F1053P0010/IITA-TMS-IBA972205 | Test |
| TMS18F1344P0100 | TMS13F1053P0010/IITA-TMS-IBA972205 | Test |
| TMS18F1344P0133 | TMS13F1053P0010/IITA-TMS-IBA972205 | Test |
| TMS18F1344P0147 | TMS13F1053P0010/IITA-TMS-IBA972205 | Test |
| TMS18F1344P0153 | TMS13F1053P0010/IITA-TMS-IBA972205 | Test |
| TMS18F1344P0159 | TMS13F1053P0010/IITA-TMS-IBA972205 | Test |
| TMS18F1344P0176 | TMS13F1053P0010/IITA-TMS-IBA972205 | Test |
| TMS18F1344P0198 | TMS13F1053P0010/IITA-TMS-IBA972205 | Test |
| TMS18F1345P0001 | TMS13F1053P0010/TMEB419 | Test |
| TMS18F1345P0012 | TMS13F1053P0010/TMEB419 | Test |
| TMS18F1345P0043 | TMS13F1053P0010/TMEB419 | Test |
| TMS18F1345P0056 | TMS13F1053P0010/TMEB419 | Test |
| TMS18F1346P0023 | TMS13F1063P0013/TMS13F1343P0002 | Test |
| TMS18F1347P0029 | TMS13F1088P0007/IITA-TMS-IBA030060A | Test |
| TMS18F1348P0024 | TMS13F1088P0007/IITA-TMS-IBA930007 | Test |
| TMS18F1348p0039 | TMS13F1088P0007/IITA-TMS-IBA930007 | Test |
| TMS18F1348P0051 | TMS13F1088P0007/IITA-TMS-IBA930007 | Test |
| TMS18F1348P0088 | TMS13F1088P0007/IITA-TMS-IBA930007 | Test |
| TMS18F1348P0092 | TMS13F1088P0007/IITA-TMS-IBA930007 | Test |
| TMS18F1348P0095 | TMS13F1088P0007/IITA-TMS-IBA930007 | Test |
| TMS18F1348P0097 | TMS13F1088P0007/IITA-TMS-IBA930007 | Test |
| TMS18F1348P0098 | TMS13F1088P0007/IITA-TMS-IBA930007 | Test |
| TMS18F1348P0101 | TMS13F1088P0007/IITA-TMS-IBA930007 | Test |
| TMS18F1348P0113 | TMS13F1088P0007/IITA-TMS-IBA930007 | Test |
| TMS18F1348P0117 | TMS13F1088P0007/IITA-TMS-IBA930007 | Test |
| TMS18F1348P0131 | TMS13F1088P0007/IITA-TMS-IBA930007 | Test |
| TMS18F1348P0156 | TMS13F1088P0007/IITA-TMS-IBA930007 | Test |
| TMS18F1348P0157 | TMS13F1088P0007/IITA-TMS-IBA930007 | Test |
| TMS18F1349P0064 | TMS13F1088P0007/IITA-TMS-IBA972205 | Test |
| TMS18F1349P0065 | TMS13F1088P0007/IITA-TMS-IBA972205 | Test |
| TMS18F1349P0123 | TMS13F1088P0007/IITA-TMS-IBA972205 | Test |
| TMS18F1349P0126 | TMS13F1088P0007/IITA-TMS-IBA972205 | Test |
| TMS18F1349P0149 | TMS13F1088P0007/IITA-TMS-IBA972205 | Test |
| TMS18F1349P0157 | TMS13F1088P0007/IITA-TMS-IBA972205 | Test |

| Accession ID | Pedigree | Entry Type |
| --- | --- | --- |
| TMS18F1350P0026 | TMS13F1088P0007/TMEB419 | Test |
| TMS18F1350P0038 | TMS13F1088P0007/TMEB419 | Test |
| TMS18F1350P0041 | TMS13F1088P0007/TMEB419 | Test |
| TMS18F1350P0056 | TMS13F1088P0007/TMEB419 | Test |
| TMS18F1350P0065 | TMS13F1088P0007/TMEB419 | Test |
| TMS18F1350P0082 | TMS13F1088P0007/TMEB419 | Test |
| TMS18F1350P0091 | TMS13F1088P0007/TMEB419 | Test |
| TMS18F1352P0002 | TMS13F1160P0005/IITA-TMS-IBA000070 | Test |
| TMS18F1352P0016 | TMS13F1160P0005/IITA-TMS-IBA000070 | Test |
| TMS18F1352P0049 | TMS13F1160P0005/IITA-TMS-IBA000070 | Test |
| TMS18F1353p0017 | TMS13F1106P0004/TMS13F1122P0005 | Test |
| TMS18F1353P0022 | TMS13F1106P0004/TMS13F1122P0005 | Test |
| TMS18F1353P0023 | TMS13F1106P0004/TMS13F1122P0005 | Test |
| TMS18F1353P0038 | TMS13F1106P0004/TMS13F1122P0005 | Test |
| TMS18F1354P0027 | TMS13F1343P0022/IITA-TMS-IBA000070 | Test |
| TMS18F1355p0025 | TMS13F1343P0022/IITA-TMS-IBA030060A | Test |
| TMS18F1355P0081 | TMS13F1343P0022/IITA-TMS-IBA030060A | Test |
| TMS18F1355P0089 | TMS13F1343P0022/IITA-TMS-IBA030060A | Test |
| TMS18F1355P0120 | TMS13F1343P0022/IITA-TMS-IBA030060A | Test |
| TMS18F1355p0038 | TMS13F1343P0022/IITA-TMS-IBA030060A | Test |
| TMS18F1359P0001 | TMS14F1201P0003/TMS14F1284P0001 | Test |
| TMS18F1361P0018 | TMS15F1041P0003/TMS15F1021P0028 | Test |
| TMS18F1361P0041 | TMS15F1041P0003/TMS15F1021P0028 | Test |
| TMS18F1364P0018 | TMS15F1143P0004/IITA-TMS-IKN130010 | Test |
| TMS18F1364P0022 | TMS15F1143P0004/IITA-TMS-IKN130010 | Test |
| TMS18F1364P0027 | TMS15F1143P0004/IITA-TMS-IKN130010 | Test |
| TMS18F1365P0005 | TMS15F1159P0006/IITA-TMS-IKN130010 | Test |
| TMS18F1365P0018 | TMS15F1159P0006/IITA-TMS-IKN130010 | Test |
| TMS18F1365P0022 | TMS15F1159P0006/IITA-TMS-IKN130010 | Test |
| TMS18F1365P0024 | TMS15F1159P0006/IITA-TMS-IKN130010 | Test |
| TMS18F1366P0012 | TMS15F1159P0006/TMS15F1021P0028 | Test |
| TMS18F1366P0014 | TMS15F1159P0006/TMS15F1021P0028 | Test |
| TMS18F1367P0030 | TMS15F1159P0006/TMS15F1305P0017 | Test |
| TMS18F1369P0014 | TMS15F1305P0017/TMS15F1021P0028 | Test |
| TMS18F1369P0015 | TMS15F1305P0017/TMS15F1021P0028 | Test |
| TMS18F1369P0023 | TMS15F1305P0017/TMS15F1021P0028 | Test |
| TMS18F1370P0008 | TMS15F1318P0009/TMS15F1041P0003 | Test |
| TMS18F1371P0001 | TMS15F1318P0009/TMS15F1305P0007 | Test |

| Accession ID | Pedigree | Entry Type |
| --- | --- | --- |
| TMS18F1371P0011 | TMS15F1318P0009/TMS15F1305P0007 | Test |
| TMS18F1372P0024 | TMS15F1329P0005/TMS15F1021P0028 | Test |
| TMS18F1372P0041 | TMS15F1329P0005/TMS15F1021P0028 | Test |
| TMS18F1373P0028 | TMS15F1329P0005/TMS15F1041P0003 | Test |
| TMS18F1377P0007 | TMS15F1396P0001/TMS15F1296P0001 | Test |
| TMS18F1377P0023 | TMS15F1396P0001/TMS15F1296P0001 | Test |
| TMS18F1380P0003 | IITA-TMS-IBA011368/IITA-TMS-IKN130010 | Test |
| TMS18F1388P0012 | IITA-TMS-IBA070337/IITA-TMS-IBA141092 | Test |
| TMS18F1389P0021 | IITA-TMS-IBA070337/TMS14F1319P0004 | Test |
| TMS18F1389P0031 | IITA-TMS-IBA070337/TMS14F1319P0004 | Test |
| TMS18F1391P0004 | IITA-TMS-IBA070593/IITA-TMS-IBA011797 | Test |
| TMS18F1395P0010 | IITA-TMS-IBA070593/IITA-TMS-IBA930007 | Test |
| TMS18F1395P0038 | IITA-TMS-IBA070593/IITA-TMS-IBA930007 | Test |
| TMS18F1395P0055 | IITA-TMS-IBA070593/IITA-TMS-IBA930007 | Test |
| TMS18F1396P0017 | IITA-TMS-IBA070593/IITA-TMS-IBA972205 | Test |
| TMS18F1396P0053 | IITA-TMS-IBA070593/IITA-TMS-IBA972205 | Test |
| TMS18F1408P0006 | IITA-TMS-IBA011797/IITA-TMS-IBA030060A | Test |
| TMS18F1409P0008 | IITA-TMS-IBA011797/IITA-TMS-IBA972205 | Test |
| TMS18F1414P0014 | IITA-TMS-IBA070337/IITA-TMS-IBA972205 | Test |
| TMS18F1414P0017 | IITA-TMS-IBA070337/IITA-TMS-IBA972205 | Test |
| TMS18F1421P0005 | IITA-TMS-IBA090516/IITA-TMS-IKN120210 | Test |
| TMS18F1421P0025 | IITA-TMS-IBA090516/IITA-TMS-IKN120210 | Test |
| TMS18F1424P0021 | IITA-TMS-IBA090516/TMEB693 | Test |
| TMS18F1426P0001 | IITA-TMS-IBA141092/IITA-TMS-IBA30572 | Test |
| TMS18F1436P0049 | IITA-TMS-IBA141092/1629MP2 | Test |
| TMS18F1436P0053 | IITA-TMS-IBA141092/1629MP2 | Test |
| TMS18F1447P0002 | IITA-TMS-IBA30572/IITA-TMS-IBA011368 | Test |
| TMS18F1447P0045 | IITA-TMS-IBA30572/IITA-TMS-IBA011368 | Test |
| TMS18F1447P0062 | IITA-TMS-IBA30572/IITA-TMS-IBA011368 | Test |
| TMS18F1447P0078 | IITA-TMS-IBA30572/IITA-TMS-IBA011368 | Test |
| TMS18F1469P0062 | TMEB419/TMS14F1319P0004 | Test |
| TMS18F1472P0042 | TMEB693/IITA-TMS-IBA030060 | Test |
| TMS18F1476P0047 | TMEB693/IITA-TMS-IKN130010 | Test |
| TMS18F1478P0047 | TMEB693/TMS13F1475P0217 | Test |
| TMS18F1480P0023 | TMEB693/TMS14F1234P0001 | Test |
| TMS18F1481P0009 | TMS13F1053P0010/IITA-TMS-IBA030060A | Test |
| TMS18F1481P0015 | TMS13F1053P0010/IITA-TMS-IBA030060A | Test |
| TMS18F1484P0036 | TMS13F1053P0010/IITA-TMS-IBA141092 | Test |

| Accession ID | Pedigree | Entry Type |
| --- | --- | --- |
| TMS18F1485P0022 | TMS14F1284P0001/TMS13F1227P0119 | Test |
| TMS18F1489P0038 | TMS13F1088P0007/TMS13F1343P0002 | Test |
| TMS18F1489P0044 | TMS13F1088P0007/TMS13F1343P0002 | Test |
| TMS18F1489P0040 | TMS13F1088P0007/TMS13F1343P0002 | Test |
| TMS18F1505P0047 | TMS13F1307P0016/IITA-TMS-IBA930007 | Test |
| TMS18F1505P0057 | TMS13F1307P0016/IITA-TMS-IBA930007 | Test |
| TMS18F1506P0018 | TMS13F1307P0016/IITA-TMS-IBA972205 | Test |
| TMS18F1520P0039 | TMS14F1201P0003/TMEB419 | Test |
| TMS18F1520P0050 | TMS14F1201P0003/TMEB419 | Test |
| TMS18F1539P0063 | TMEB419/TMS14F1073P0001 | Test |
| TMS18F1539P0073 | TMEB419/TMS14F1073P0001 | Test |
| TMS18F1562P0023 | IITA-TMS-IBA071313/IITA-TMS-IBA930007 | Test |
| TMS18F1562P0025 | IITA-TMS-IBA071313/IITA-TMS-IBA930007 | Test |
| TMS18F1562P0026 | IITA-TMS-IBA071313/IITA-TMS-IBA930007 | Test |
| TMS18F1562P0030 | IITA-TMS-IBA071313/IITA-TMS-IBA930007 | Test |
| TMS18F1562P0055 | IITA-TMS-IBA071313/IITA-TMS-IBA930007 | Test |
