# Supplementary material for: Genome-wide association study identifies novel genes for plant architecture and yield traits in cassava (Manihot esculenta Crantz)
Source: Front Plant Sci. 2025 Sep 10;16:1660789. doi: 10.3389/fpls.2025.1660789 (PMC12457381; doi:10.3389/fpls.2025.1660789)
Supplement: Supplementary file 2 [file Table2.docx]

**Table S2: Details of the commercial check varieties**

| Variety | Yield (t/ha) | Dry Matter Content (%) | Starch Content (%) | Physiology | Other Features |
| --- | --- | --- | --- | --- | --- |
| TMEB419  (TME419) | 36 | 40 |  | Erect, straight type excellent for mechanization | Green petiole, light brown stem, light/cream root, CMD resistance, low cyanide potential, Good for starch, flour, garri, and fresh consumption |
| IITA-TMS-IBA000070  (BABA-70) | 37.5 | 38.5 | 28 | Compact plant type, light brown stem, and red petiole color | High yielding and early bulking, good weed control, moderate dry matter content, resistant to Cassava Mosaic Disease (CMD), excellent for gari and fufu |
| IITA-TMS-IBA98058  (Dixon) | 35 | 35 |  | Erect plant type, excellent drought tolerance | Red petiole, silver-green stem, white root, CMD and CGM resistant, drought tolerant, Good for garri production |
| NR130124  (HOPE) | 40.1 | 33.2 | 24.3 | Compact branching pattern | Resistant to Cassava Mosaic Disease (CMD), cassava anthracnose disease (CAD), cassava mealybug (CM), cassava bacterial blight (CBB), and cassava green mite (CGM). Good for mechanization and has high fresh root yield, Excellent for gari and fufu production |
| IITA-TMS-IBA30572 | >25 | 25 |  | Compact structure with good branching habits | Moderate CMD resistance, early bulking, high starch and high yielding |
| IITA-TMS-IBA982101 | >25 | 32 |  | Compact structure  with good branching  habits | |

Source: (BASICS-II, 2020; *Released Cassava Varieties in Nigeria | Cassava Seed Tracker*)
