# Supplementary material for: Genome-wide association study identifies novel genes for plant architecture and yield traits in cassava (Manihot esculenta Crantz)
Source: Front Plant Sci. 2025 Sep 10;16:1660789. doi: 10.3389/fpls.2025.1660789 (PMC12457381; doi:10.3389/fpls.2025.1660789)
Supplement: Supplementary file 3 [file Table3.docx]

| **Table S3: Plant Architecture Traits** | |
| --- | --- |
| Traits | Mode of Scoring |
| Stem diameter (STMDI9) (mm) | Measured using digital vernier caliper |
| Shoot weight (SHTWT) (kg) | Total weight of aboveground biomass (stems and leaves) per plot at harvest |
| Plant height at 9 months after planting (PLTHT9) (cm) | Measured using meter rule |
| Number of lodged plants per plot (LODG) | Count |
| Branching habit at 9 months after planting (BRNHB9) | Categorical Scoring |
| Angle of branching (ANGBR9) (0) | Measured using digital compass |
| Number of plants per stand (PPSTD9) | Count |
| Height at first branch at 9 months after planting (BRNHT9) (cm) | Measured using meter rule |
| Plant height at 6 months after planting (PLTHT6) (cm) | Measured using meter rule |
| Top yield (TYLD) (t/ha) | Estimate: (SHTWT/(MaxNumberHarvested) * 12000/1000) |
| Height at first branch at 6 months after planting (BRNHT6) (cm) | Measured using meter rule |

| Yield-Related Traits | |
| --- | --- |
| Traits | Mode of Scoring |
| Fresh root yield (FYLD) (t/ha) | Estimate: (RTWT/(MaxNumberHarvested) * 12000/1000) |
| Dry yield (DYLD) (t/ha) | Estimate (FYLD*(DM/100)) |
| Starch content (SC) (%) | Estimate: Specific gravity (SG) method (210.8 * SG -213.4) |
| Dry matter content (DM) (%) | Estimate: Specific gravity (SG) method (158.3 * SG -142) |
| Number of harvested roots (RTNO) | Count |
| Fresh root weight (RTWT) (kg) | Total weight of fresh roots harvested per plot at harvest |
| Harvest index (HI) | Estimate (Roots weight / Roots weight + Shoot weight) |
